# Targetable Mechanical Properties by Switching between Self‐Sorting and Co‐assembly with *In Situ* Formed Tripodal Ketoenamine Supramolecular Hydrogels

**DOI:** 10.1002/cnma.201800198

**Published:** 2018-06-22

**Authors:** Jamie S. Foster, Andrew W. Prentice, Ross S. Forgan, Martin J. Paterson, Gareth O. Lloyd

**Affiliations:** ^1^ Institute of Chemical Sciences, School of Engineering and Physical Sciences Heriot-Watt University William Perkin Building Edinburgh Scotland, United Kingdom EH11 4AS; ^2^ WestCHEM, School of Chemistry University of Glasgow Joseph Black Building, University of Glasgow, University Avenue Glasgow United Kingdom G12 8QQ.

**Keywords:** supramolecular gel, self-sorting, co-assembly, dynamic covalent chemistry, nanofibers

## Abstract

A new family of supramolecular hydrogelators are introduced in which self‐sorting and co‐assembly can be utilised in the tuneability of the mechanical properties of the materials, a property closely tied to the nanostructure of the gel network. The *in situ* reactivity of the components of the gelators allows for system chemistry concepts to be applied to the formation of the gels and shows that molecular properties, and not necessarily the chemical identity, determines some gel properties in these family of gels.

## Introduction

The research volume concerning supramolecular hydrogels formed utilising low molecular weight gelators (LMWGs) has increased dramatically in recent years.^1^ This can largely be attributed to the plethora of applications they can be utilised for, such as organic electronics^2^, cell growth, and drug delivery, to name but a few.^3^ Established examples of LMWGs are chemically diverse and include species such as benzene‐1,3,5‐triamide (BTA) derivatives,^4^ functionalised amino acid based systems^5^ and gelators derived from carbohydrates,^6^ again to name but a few. In order to form a gel the gelator compounds are dissolved in the appropriate solvent before a gelation trigger is applied. Examples of triggers include sonication^7^ or a change in the solvent conditions, such as temperature and pH.^8^ More recently the chemical reactivity between two or more components has been used to induce gelation; these reactions occur *in situ,* gelling the solvent in which the reaction takes place.^9^ It has already been demonstrated that discotic, tripodal molecules with *C_3_* symmetry can act as effective gelators.^10^ These gelators all follow the same design principles that dictate a central core unit surrounded by three identical/similar and equally spaced peripheral ‘leg’ units. The discotic, anisotropic nature of these molecules allows efficient face‐to‐face stacking driven by supramolecular interactions i. e. π‐π stacking, hydrogen bonding and other dispersion forces. It is these interactions between the central core of the gelating molecules that promote fibre growth while the physical properties of the gel can be altered through changes to the chemical composition of the leg units. Supramolecular gels are classically thought of as containing a single LMWG, this however does not need be the case. Recent work has demonstrated that multi‐component gels, particularly those formed using two different LMWGs, are entirely feasible.^11^ When considering the network assembly of multi‐component gels two concepts must take precedence, those of self‐sorting and co‐assembly.

In a self‐sorting system, each of the component gelators arrange themselves so that the fibres of the gel network assemble containing only one gelator or the other. A co‐assembly system describes a gel where the component gelators interdigitate with each other when forming the gel fibres in either a statistical or ordered manner. Another theoretical possibility is a self‐sorting/co‐assembly hybrid where self‐sorting of the monomers into individual component fibres which subsequently co‐assembly into the fibrils results in the solvent spanning gel network, i. e. there are not two networks, which would be the case of a double network formed by a fully self‐sorted system. The close structural relation between the family members described below has allowed our exploration of the concepts of both self‐sorting^12^ and co‐assembly^13^ coupled with *in situ* reaction chemistry.^14^

The reaction between the *C_3_* symmetric trialdehyde 1,3,5‐triformylphloroglucinol (***A***) and a variety of amines has already been shown to produce discotic molecules.^15^ The product is an imine (Schiff base) which can undergo keto‐enol tautomerisation; the keto form being thermodynamically favoured.^16^ These discotic molecules have been shown to stack in a columnar fashion through their demonstrated ability to form liquid crystals.15a

**Figure 1:**
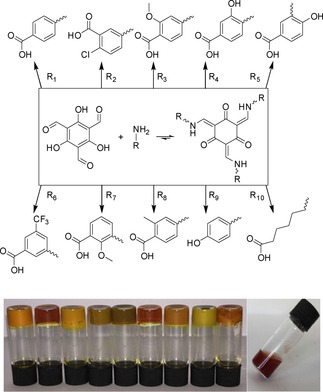
Schematic representation of the ten compounds synthesised and isolated from an *in situ* reaction at high pH after the addition of glucono‐delta‐lactone (**GdL**). Photo below shows the gels resulting from an *in situ* preparation method (gels R_1_ to R_9_ left to right). Photo on far right shows precipitate formed when R_10_ is used.

Herein, we present a family of ketoenamine based hydrogelators (Figure 1). These gelators can be formed both *in situ* by dissolving ***A*** and the desired amine in water, and *ex situ* by refluxing ***A*** with the appropriate amine in ethanol, so that we can utilise the reactivity as part of the materials synthesis by doing *in situ* experiments of mixtures. The combination of ***A*** with eight aminobenzoic acids and a phenolic species has produced a family of nine distinct hydrogels as described in the main manuscript (Figure 1). Chemical analysis has shown that regardless of whether the gelator is formed *in situ* or *ex situ*, the product of the reaction is the same. In all cases, ^1^H NMR spectroscopy has shown the compounds are in the thermodynamically more favoured ketoenamine tautomer (as opposed to the enol‐imine tautomer).

## Results and Discussion

### Pure Gelators

Gelation is triggered through a reduction in the pH of the reaction solution. When setting a gel the gelator compounds are first dissolved in water at a pH above their apparent p*K*_a_. In order to ensure the highest degree of structural homogeneity in the gel the pH was lowered using glucono‐delta‐lactone (**GdL**) which resulted in a uniform lowering of the pH.^17^ The exception to this method of setting is gel formation by R_9_ which features hydroxyl groups on the peripheral legs. In order to set this gel concentrated hydrochloric acid must be used to rapidly lower the pH and trap out the metastable gel state. This results in the lack of a homogenous structure that can be clearly observed in Figure 1, as well as reflected in the significantly lower *G′* and ‘*yield stress*’ when compared to the carboxylic acid appended species.

**Figure 2:**
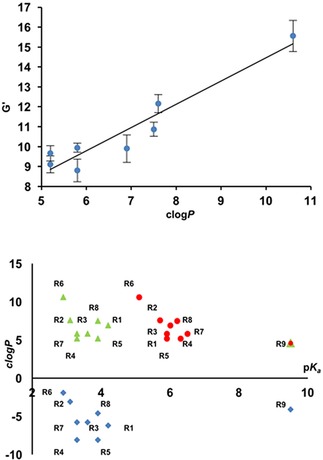
Top graph shows *c*log*P* values (hydrophobic character) plotted against *G′* values for the gels (mechanical properties) showing a clear trend between the members of the gel family. Line added to guide the eye. Bottom graph shows the calculated values for the p*K_a_* vs *c*log*P* of gelators R_1_ to R_9_. *c*log*P* vs calculated p*K_a_* (green Δ) neutral species, *c*log*P* vs calculated p*K_a_* (blue ◊) trianionic species and *c*log*P* vs measurement p*K_a_* (red ○).

The exact pH at which gelation occurs is an important physical property of any gelator that operates by means of a pH trigger. The fact that this particular family of gelators is versatile in terms of the aminobenzoic acids used allowed us to raise the notion that the pH of gelation (the apparent p*K*_a_ of the gelator) could be controllably altered. This alteration of gelation pH is dependent on the modification to the p*K_a_* of the gelator's carboxyl or hydroxyl groups (i. e. electron removing and adding substitutions on the aromatic ring) and the hydrophobicity^18^ (related to the ease at which the compounds self‐assemble). The apparent p*K_a_* for the family of presented gelators was determined^19^ in order to ascertain the pH required to induce gelation. As can be seen in Figure 2, differences in the apparent p*K_a_* of the gelators are related to the calculated *c*log*P* values (hydrophobicity) and calculated p*K_a_* values from the gelator structures.

These relationships have been noted in previous work concerning dipeptide and BTA gel systems, and can be attributed to the intrinsic increase in hydrophobicity that arises from the supramolecular assembly process that causes gelation. The hydrophobicity of the protonated species, which we define here as their calculated *c*log*P* values, would appear to be related to the mechanical properties of the resultant gels.^18^,^20^ In the case of gels R_2_ and R_6_, they show the highest degree of mechanical strength which coincides with the highest *c*log*P* and therefore hydrophobic character. The clear relationship between the hydrophobic character for the neutral molecules and the *G′* mechanical property of the corresponding gels is shown in Figure 2.

Another observation is the difference in transparency between the gels (Figure 1). Gels R_2_ and R_6_ are noticeably more transparent than the other gels. This transparency is a result of gels R_2_ and R_6_ having narrower fibres and therefore scattering less light (Figure S59). Gels R_2_ and R_6_ also demonstrate the two lowest CGC values. The responses of the nine presented gels to mechanical stimulus were determined using rheometry (Table 1). Although differences in mechanical properties are apparent when comparing one gel to another the difference between the same gel prepared *ex situ* and *in situ* is negligible (see ESI). Once a constant value for *G′* had been recording during the time sweep experiments, the gelatinous nature of the materials was confirmed using frequency sweep rheometry.

**Table 1:** Physical Properties of the gels at 40 mM gelator concentration. Standard deviation errors are shown in brackets, values rounded to the nearest 100 for the G′ and G′′, and 10 for the yield stress.

Gel	G′ (Pa)	G′′ (Pa)	Yield stress (μNm)	CGC (Wt%)	Apparent p*K_a_*
R_1_	9000(200)	2300(100)	140(10)	0.3	6.1–5.8
R_2_	12700(100)	3300(200)	80(20)	0.2	5.8–5.5
R_3_	10200(300)	2100(100)	140(20)	0.5	6.1–5.7
R_4_	9300(200)	2100(200)	40(10)	0.4	6.5–6.2
R_5_	10800(200)	3100(400)	80(20)	0.4	6.1–5.8
R_6_	15400(300)	2600(200)	250(30)	0.1	5.2–5.0
R_7_	8800(200)	2100(300)	90(30)	0.5	6.5–6.3
R_8_	9600(100)	2500(200)	70(20)	0.5	6.4–6.0
R_9_	1200(100)	300(300)	20(40)	0.9	9.9–9.0

**Figure 3:**
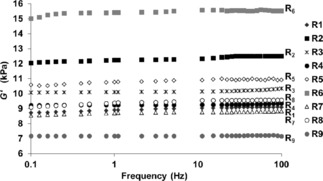
*G′* frequency sweeps from 0.1–100 Hz for gels R_1_–R_9_ showing the varying mechanical properties of the gels at a concentration of 40 mM.

For all gels the *G′* and *G“* values remained constant over a frequency range of 0.1–100 Hz, with the value of *G′* several times the value recorded for *G”* (Figure 3). To examine the gels’ non‐linear rheological behaviour stress sweep experiments were conducted. The defining point of the experiment is the *yield stress*, below which *G′* remains essentially constant, but above which the gel begins to flow (see ESI).

In order to ascertain the nature of the supramolecular structure and assembly process of the gels, variable concentration rheology experiments were conducted using gelator R_1_ as an exemplar. The values for *G′* and “yield stress”, over a range of concentrations from 40 mM to 400 mM, show relationships such that gel concentration of *G′* ∝ *concentration* ^*1.9*^ and “yield stress” ∝ *concentration* ^*1.6*^. These relationships are in agreement with the cellular solid model description of a gel which predicts relationships of *G′* ∝ *concentration* ^*n*^ and “yield stress” ∝ *concentration* ^*n*^ (where *n* normally varies between 1 and 2)^18^,^20^,^21^ as opposed to the alternative colloidal gel description. The cellular model describes a material that derives its strength from its composition of load bearing struts that are interconnected by crosslinks, which deform by bending.^22^ Considering the results of the variable concentration rheology experiments, calculated Avrami constants (same as Fractal dimensions, see SI for details on how these constants were determined)^22^ and the morphology of the gels determined with scanning electron microscopy (SEM), there is a clear suggestion of a nucleation event followed by the growth of high aspect ratio fibres involved in the gel assembly.

Powder diffraction (PXRD) patterns of the xerogels produced by drying the nine presented gels show there is a characteristic broad reflection corresponding to a *d* spacing of 3.34–3.39 Å, a value that is very similar to the BTA supramolecular gels4a and also the tris(*N*‐salicylideneanilines) produced by Yelmaggad *et. al*.15a This distance can be attributed to the stacking distance between the core units of the individual gel molecules in the same fibre. The results of the diffraction experiments show that the stacking pattern is likely to be supramolecular in nature with the key interaction being the π‐π stacking.

### Mixed Gelators

With this knowledge of the molecular self‐assembly in‐hand we aimed to show that co‐assembly and self‐sorting could be utilised to controllably alter the mechanical properties of the materials. Connected to this is the *in situ* reactivity. Using concepts well developed in systems chemistry and combinatorial dynamic covalent chemistry libraries we would expect thermodynamically controlled statistic mixtures of products when mixtures of core are reacted with two or more leg reactants.^23^ We thus studied the resultant materials from reactions between R_1_, R_6_ and R_10_. R_1_ forms one of the weaker gels mechanically, R_6_ is the strongest gelator mechanically, and R_10_ shows no gelation and crystallisation instead.

**Figure 4:**
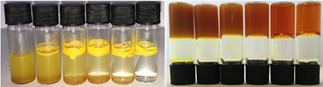
Vials containing an *ex situ* prepared gelator (from left to right) containing 0, 20, 40, 60, 80 and 100% R_6_ and 100, 80, 60, 40, 20 and 0% R_1_ as the ‘leg’ units of the tripodal gelators for a total gelator concentration of 47 mM. Left is shown the gelator molecule mixtures in pH 7 water showing the different hydrophobic characteristics and right is shown the gels formed after raising the pH to dissolve gelator mixtures and setting utilising **GdL**.

Investigations of this hypothesis were conducted with gelators R_1_ and R_6_ owing to their widely different mechanical strengths and apparent p*K*_a_ (Table 1). Mixtures of the two compounds in a variety of ratios were prepared at constant core concentration and the gels were set using the previously outlined method (both *in situ* and *ex situ*, where both sets of experiments matched). Initial visual inspection found gels with strikingly different appearances. The appearance of the gels followed the trend of opaque to transparent as the concentration of R_6_ was increased and R_1_ was decreased; while the total gelator concentration remained constant (Figure 4).

**Figure 5:**
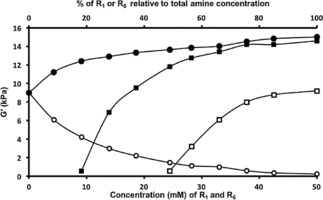
Graph showing the G′ of gels prepared with R_1_, 4‐aminobenzoic acid and an additional amine, either R_6_ 3‐amino‐5‐(trifluoromethyl)benzoic acid (filled •) or R_10_ 6‐aminohexanoic acid (empty ○). The upper X‐axis shows the percentage of components within the constant total amine concentration. Lower X axis and addition set of graphs (R_1_, 4‐aminobenzoic acid (filled ▪) and R_6_ 3‐amino‐5‐(trifluoromethyl)benzoic acid (empty □)) presents data of the concentration vs G′ of gels of the two amines. This indicates the initial and final G′ values of the mixed gels either represents the G′ values of R_1_ or R_6_/R_10_.

UV‐Vis transmission experiments were used to characterise the transparency of the mixed gelator systems (Figure S59), and this confirmed the visual observations. In terms of physical mechanical strength, this also increases with transparency which, as previously demonstrated, is related to the hydrophobicity of the supramolecular monomer units (Figure 5). The hydrophobicity of the undissolved mixed gelator system can be seen in Figure 4. In order to differentiate the possible assembly processes of the fibres, one of self‐ or co‐assembly, the apparent p*K*_a_ of the mixed systems was determined (See SI for details). The pH plateau region shifts from the value recorded for R_1_ to the value for a R_6_.

The fact that the change is gradual would suggest a mixing effect and the concept that both components contribute *pro rata* to the overall apparent p*K_a_* of the solution. It is important to observe the consistence in the rate of change of the plateau region suggesting an averaging of protonation across both types of gelator, giving rise to the idea that this mixed gel system forms through a co‐assembly process. Two plateau regions would suggest a stepwise protonation process where the gelators are protonated independently of each other giving rise to a self‐sorted system. ^1^H solution NMR spectroscopic experiments were used to confirm the co‐assembly of the mixed gelator system containing R_1_ and R_6_ after the addition of **GdL**.11a,11c,11j,11k–11n,13a The experiment was conducted in D_2_O using a 50 : 50 mix (by concentration) of the gelators with spectra recorded every five minutes for two hours. When examining the integrations (relative concentrations) for each of the molecules, a simultaneous decrease in the signal intensity was observed. This is indicative of a co‐assembly process where both gelator molecules enter the solid phase of the gel network becoming NMR invisible. PXRD analysis of dried samples of the mixed gels show a shift of the characteristic stacking peak from ∼3.53 Å to ∼3.32 Å. This can be interpreted as a change in the average stacking distance between the discotic molecules that form the fibres. This shift in the peak is further evidence of supramolecular co‐assembly. A broadening or appearance of a second peak would be suggestive of a system that was self‐sorting in nature.

The experiments described so far for this mixed gel system of R_1_:R_6_ were performed on *ex situ* synthesised pure gel compounds. To further investigate the chemistry of these compounds and the physical properties, *in situ* reactions were performed to generate the gels of mixed components. These *in situ* derived materials were found to be analytically (rheology, visual appearance, morphology) identical to those materials made using the *ex situ* compounds. However, chemical analysis of the compounds produced by the *in situ* methods showed that the materials are different chemically. HPLC traces of the product mixtures reveal four compounds produced during the *in situ* reaction. To explain this observation we need to describe the reaction between the core trialdehyde and amino periphery species.

The first step of the reaction is the formation of imine groups. Upon formation of the complete set of three imine groups, the enol form will undergo a tautomerisation to the keto form. The imine stage of the reaction should behave as a typical dynamic covalent chemistry mixture, in that it should form four species with a thermodynamically controlled statistic distribution: 3:0 R_1_:R_6_; 2 : 1 R_1_:R_6_; 1 : 2 R_1_:R_6_; 0 : 3 R_1_:R_6_ (Figure S70). We can assign two of the four species in the HPLC traces to the pure 3:0 R_1_:R_6_ and 0 : 3 R_1_:R_6_ species and determine their abundance. The calculated concentrations of 3:0 R_1_:R_6_ and 0 : 3 R_1_:R_6_ account for 28% and 24% of the total concentration of the reaction products, indicating that it is likely the dynamic covalent chemical distribution for the reaction is 1 : 1 : 1 : 1. The *ex situ* reactions cannot redistribute from 1:0:0 : 1 to the 1 : 1 : 1 : 1 mixture as the keto‐enol tautomerisation is non‐reversible for these compounds.

We feel this is an interesting result, in that a two component chemical mixture is giving the same rheological material properties as a four component chemical mixture. This type of scientific insight would not be possible without the opportunity to mix the chemical functionalities on the periphery of the gel molecules utilising the dynamic covalent chemistry. Being able to then fix that distribution of the functionality utilising the tautomerisation results in a chemically diverse set of compounds. This diversity, however, results in the same gel properties in the form of the transparent character and rheological mechanical properties. This means that the average chemical properties of the compounds, and not the properties of the individual compounds, determine the materials properties in this family of compounds.

When a mixed gel system was produced using R_1_ and R_10_ a decrease in mechanical strength was observed when the concentration of R_10_ is increased relative to R_1_ for a constant total concentration of potential gelator (i. e. the core concentration is constant). R_10_ is a non‐gelator; its addition to the gel system when the total ligand concentration remains constant results in a reduction in the concentration of ‘gelation active’ ligands. Whereas the R_1_, R_6_ mixed system demonstrates a co‐assembly process, the R_1_, R_10_ system shows an ability to self‐sort (this can also be described as orthogonal assembly).^24^ Specifically, the system sorts into gelating (R_1_) and non‐gelating crystalline phases (R_10_) (see SI for details of PXRD, p*K*_a_ and NMR spectroscopic experiments).

The *in situ* preparation of an R_1_ R_10_ mixed system using 1.5 equivalents of each of the amines relative to the core **A** produces not just a *supramolecular* self‐sorted system but also a *chemically* self‐sorted system. There is no evidence of a statistical mixture of R_1_, R_10_, and the asymmetric ligands featuring 2 : 1 R_1_:R_10_ and 1 : 2 R_1_:R_10_ units (Figure S71). Chemical analysis indicates purely R_1_ and R_10_, resulting in the *ex situ* and *in situ* experiments matching, in contrast to the R_1_:R_6_ gelation mixed system. Below we explain these observations of dynamic covalent chemistry differences between R_1_:R_6_ and R_1_:R_10_.

### Computational Section

The ground state energetic pathway of various systems containing R_1_, R_6_, R_10_ and R_11_ subunits (R_11_ is the methyl derivative that was only investigated computationally as it reduced the conformational flexibility of the alkyl chains as found in R_10_), with respect to the central core *A*, were explored using density functional theory, as implemented in Gaussian 09 (Revision D.01).^25^ All structures were optimised in the gas phase and further validated to be true minima via analytical Hessian computation, using both the B97‐D^26^ and B3LYP^27^ functionals with a polarized split valence double‐ζ basis, 6‐31G(d,p).

For the individual tripodal keto‐enamine systems there are two possible conformations available, the C_3_ and C_s_ arrangement, both of which are observed experimentally in solution utilising NMR. We find the C_3_ and C_S_ conformations to be practically isoenergetic, with differences of ∼1 kcal mol^−1^ across the various combinations of R_1_, R_6_, R_10_ and R_11_ (cf. Table S3).

The overall reaction energetics of the systems was then examined; we have provided the results pertaining to the C_3_ arrangement only (see Table 2) as analogous trends were observed for the C_s_ conformation. The overall reaction energetics, quoted both in terms of electronic and zero point corrected energies, were based upon the stoichiometric reaction between ***A*** and the amine of choice, generating the tripodal keto‐enamine system and water. The general trend showed that upon replacement of a 4‐aminobenzoic acid subunit to that of 6‐aminohexanoic acid or methylamine, the overall reaction exothermicity was found to increase in a stepwise fashion. This indicates that under dynamic covalent reaction conditions the addition of the alkyl amino functionality is favoured over that of the aromatic amino. When comparing the replacement of a 4‐aminobenzoic acid to the alternative aromatic system, 5‐amino‐3‐(trifluoromethyl)benzoic acid, the nature of the overall reaction is reasonably unchanged. This agrees well with the experimental observations.

**Table 2:** Computed electronic and zero point corrected reaction energetics for the formation of various tripodal keto‐enamine systems. All values reported pertain to the *C*_3_ conformation and are in kcal mol^−1^.

	B3LYP		B97‐D	
Structure	Electronic	Zero Point Correct.	Electronic	Zero Point Correct.
3R_1_	7.104	2.711	−0.022	−3.423
2R_1_:1R_10_	0.528	−4.009	−6.052	−9.569
1R_1_:2R_10_	−5.417	−10.120	−11.338	−15.082
3R_10_	−10.636	−15.469	−15.829	−19.918
2R_1_:1R_11_	1.407	−2.779	−4.763	−7.947
1R_1_:2R_11_	−3.755	−7.644	−8.967	‐11.891
3R_11_	−8.179	−11.789	−12.393	−14.957
2R_1_:1R_6_	7.616	3.038	0.393	−3.203
1R_1_:2R_6_	8.079	3.397	0.789	−2.953
3R_6_	8.596	3.788	1.084	−2.706

As ***A*** undergoes subunit addition in the enol form, with keto‐enol tautomerism occurring when the addition of the three subunits is complete, we further explored the reaction energetics of each separate addition process. For the mixed systems we have taken the approach of initially adding the same subunit twice, followed by the addition of the remaining unit. In brief, it was found that the addition of the alkyl functional groups resulted in reactions with lower endothermicities, and in some cases exothermic reactions in nature, when compared to the initial R_1_. When we investigate the addition of R_6_ we see no apparent difference (cf. Tables S4–S5).

Finally, we show the energetic difference between the enol‐imine and keto‐enamine forms of the three‐subunit systems all in the C_3_ conformation. We find the keto‐enamine to be significantly more stable with respect to the enol‐imine form (Table S6). This again agrees well with the experimental observation of only finding the keto‐enamine form of the compounds studied, and also in agreement with previous computational calculations.^16^

## Conclusions

In conclusion, we have presented a versatile family of *C*_3_ symmetric ketoenamine based hydrogelators that have shown the ability to have many of their gelation properties selectively tuned. These properties include their mechanical properties, apparent p*K*_a_, transparency and colour. We have utilised the *in situ* reactivity of the family to show that co‐assembly and self‐sorting are possible in terms of the supramolecular assembly of the materials and chemical reactivity. This gives the ability to pick between co‐assembly and self‐sorting to tune the full scale of mechanical properties of the materials and to highlight that certain chemical properties as a collective, rather than the individual chemical entities, can dictate some gel properties. In a specific case, we highlight the relationship between hydrophobic character and gel rheological mechanical properties, which is partly related to the size of the nanofibers that make up the gel solid network.

## Conflict of interest

The authors declare no conflict of interest.

## Supporting information

As a service to our authors and readers, this journal provides supporting information supplied by the authors. Such materials are peer reviewed and may be re‐organized for online delivery, but are not copy‐edited or typeset. Technical support issues arising from supporting information (other than missing files) should be addressed to the authors.

Supplementary
