# Supplementary material for: Targetable Mechanical Properties by Switching between Self‐Sorting and Co‐assembly with In Situ Formed Tripodal Ketoenamine Supramolecular Hydrogels
Source: ChemNanoMat. 2018 Jun 22;4(8):853–9. doi: 10.1002/cnma.201800198 (PMC6473556; doi:10.1002/cnma.201800198)
Supplement: Supplementary file 1 — Supplementary [file CNMA-4-853-s001.pdf]

Supporting Information

© Copyright Wiley-VCH Verlag GmbH & Co. KGaA, 69451 Weinheim, 2018

Targetable Mechanical Properties by Switching between Self-Sorting and Co-assembly with *In Situ* Formed Tripodal Ketoenamine Supramolecular Hydrogels

Jamie S. Foster, Andrew W. Prentice, Ross S. Forgan, Martin J. Paterson, and Gareth O. Lloyd*
© 2018 The Authors. Published by Wiley-VCH Verlag GmbH & Co. KGaA. This is an open access article under the terms of the Creative Commons Attribution License, which permits use, distribution and reproduction in any medium, provided the original work is properly cited.This manuscript is part of a Special Issue on Supramolecular Nanostructures. .

Targetable mechanical properties by switching between self-sorting and co-assembly with *in-situ* formed tripodal ketoenamine supramolecular hydrogels

Jamie S. Foster,[a] Andrew W. Prentice,[a] Ross S. Forgan,[b] Martin J. Paterson,[a] and Gareth O. Lloyd[a*]

[a] Institute of Chemical Sciences, School of Engineering & Physical Sciences, Heriot-Watt University, Edinburgh, United Kingdom, EH11 4AS.
[b] *WestCHEM* School of Chemistry, Joseph Black Building, University of Glasgow, University Avenue, Glasgow, United Kingdom, G12 8QQ.

Supporting Information

Contents

1.0 Technical Details 5
1.1 Chemical list 5
1.2 Powder X-Ray Diffraction: 5
1.3 NMR: 5
1.4 SEM: 5
1.5 IR: 5
1.6 Rheology: 6
1.6.1 Time sweep experiments: 6
1.6.2 Frequency sweep experiment 6
1.6.3 Stress sweep experiments: 6
1.8 pH meter: 6
1.9 Mass Spectrometery: 6
2.0 Synthetic Details and Compound Analysis 7
2.1 Synthesis of 1,3,5-triformylphloroglucinol (A) 7
2.1.1 Yield: 7
2.1.2 ^{1}H NMR 7
2.1.3 ^{13}C NMR 7
2.1.4 Elemental analysis 7
2.1.5 Melting point 7
2.2 *Ex Situ* Synthesis of gelators R_1 to R_9 inclusively 8
2.3 Analysis of *ex situ* prepared gelators R_1 to R_9 inclusively 9
2.3.1 Gelator R_1 9
2.3.2 Gelator R_2 10
2.3.3 Gelator R_3 11
2.3.4 Gelator R_4 12
2.3.5 Gelator R_5 13
2.3.6 Gelator R_6 14
2.3.7 Gelator R_7 15
2.3.8 Gelator R_8 16
2.3.9 Gelator R_9 17

2.4 Synthesis of compound R_{10} 18
2.5 Chemical Analysis notes 19
3.0 Methods for hydrogel Preparation. 20
3.1 Setting gel using *In situ* gel preparation method (gels R_1 to R_8 inclusively): 20
3.2 *In situ* R9 gel formation: 20
3.3 Setting gel using the *ex situ* prepared compound (gels R_1 to R_8 inclusively): 21
3.4 Setting gel R9 using the *ex situ* prepared compound: 21
3.5 Critical gel concentration (CGC): 21
4.0 Morphology determination of single amine Gels 22
4.1 Gel R_1 22
4.2 Gel R_2 22
4.3 Gel R_3 23
4.4 Gel R_4 23
4.5 Gel R_5 23
4.6 Gel R_6 24
4.7 Gel R_7 24
4.8 Gel R_8 24
4.9 Gel R_9 25
5.0 Rheological studies of single amine gels 26
5.1 Rheological characterisation of *in situ* and *ex situ* prepared gel R_1 26
5.2 Rheological characterisation of *in situ* and *ex situ* prepared gel R_2 27
5.3 Rheological characterisation of *in situ* and *ex situ* prepared gel R_3 28
5.4 Rheological characterisation of *in situ* and *ex situ* prepared gel R_4 29
5.5 Rheological characterisation of *in situ* and *ex situ* prepared gel R_5 30
5.6 Rheological characterisation of *in situ* and *ex situ* prepared gel R_6 31
5.7 Rheological characterisation of *in situ* and *ex situ* prepared gel R_7 32
5.8 Rheological characterisation of *in situ* and *ex situ* prepared gel R_8 33
5.9 Rheological characterisation of *in situ* and *ex situ* prepared gel R_9 34
6.0 Apparent pK_as for synthesised gelators R_1 – R_9 37
7.0 PXRD Patterns for dried gel samples R_1 – R_9 42
7.1 PXRD pattern of non gelator R_{10} 43
8.0 Analysis of mixed gelators systems. 44
8.1 UV-vis transmission data recorded for various gels of R_1 and R_6 mixtures 44
8.2 PXRD analysis gels of R_1 and R_6 mixtures 45

8.3 Morphology determined by SEM for gels of R_1 and R_6 mixtures....................47
8.4 Apparent pK_a of R_1 and R_6 mixtures....................48
8.5 NMR of R_1 : R_6 gelator co-assembly process....................49
8.6 PXRD analysis of xerogels of R_1 and R_{10} 50:50 mixture....................50
8.7 Apparent pK_a of R_1 and R_{10} mixture....................51
8.8 NMR of R_1 : R_{10} gelator self-sorting process....................52
8.9 HPLC of R1 : R6 gelator process....................52
9.0 Computational Calculations....................54

1.0 Technical Details

All references in this SI document refer to the reference list of the main manuscript.

1.1 Chemical list

Chemicals were obtained from the following and used as supplied.

Trifluoroacetic acid – **Fluorochem**

Hexamethylenetetramine, Glucono-*delta*-lactone (**GdL)**, 4-amino-2-methoxybenzoic acid, 5-amino-2-chlorobenzoic acid, 4-amino-2-methylbenzoic acid, 4-amino-3-hydroxybenzoic acid – **Alfa Aesar**

Phloroglucinol, 6-aminohexanoic acid, 4-aminobenzoic acid, 3-amino-2-methoxybenzoic acid, 3-amino-4-hydroxybenzoic acid, 4-aminophenol – **Sigma Aldrich**

4-amino-2-fluorobenzoic acid, 3-amino-5-(trifluoromethyl)benzoic acid – **Apollo Scientific**

Deuterium oxide (D_2O), d_6-dimethylsulfoxide (d_6-DMSO) – **Cambridge isotope laboratories**

Dichloromethane (DCM), ethanol. sodium hydroxide pellets, hydrochloric acid **– Fisher Chemical**

1.2 Powder X-Ray Diffraction:

PXRD patterns were collected at room temperature using a Bruker D8 Advance powder diffractometer in reflectance mode. Lynxeye super speed detector was used with the radiation being monochromated Cu Kα1, with a characteristic wavelength of 1.541 Å. 30 minute scans over the range $5° \leq 2\theta \leq 60°$ (stepsize = 0.014481°/counting time = 0.5 s/steps).

1.3 NMR:

NMR spectra were recorded on a Bruker AV 400 operating at 400.1 MHz for ^{1}H, 282.4 MHz for ^{19}F and 100.6 MHz for ^{13}C experiments. All spectra were recorded using 10 -15 mg of sample in 0.6 ml of solvent. Spectra recorded in D_2O also feature the addition of NaOH (approximately 5 mg) to dissolve the sample unless otherwise stated.

1.4 SEM:

SEM images were produced using a Philips XL30 LaB6 ESEM equipped with an Oxford Instrument X-max 80 EDX detector at 3 kV. Wet gel samples were placed on carbon sticky tabs mounted on aluminium SEM stubs. Once mounted, samples were dried under dynamic vacuum (< 10 mBar) in a desiccator for 48 hours. These dried samples were given a thin coating of gold, to prevent charging effects during SEM imaging, by placing samples in a sputter coater for two minutes at 12 μA.

1.5 IR:

IR spectra were recorded on a Nicolet is5 instrument using 24 scans at a resolution of 1 cm^{-1} and data spacing of 0.964 cm^{-1}.

1.6 Rheology:

Rheological experiments were performed on a Bohlin nano II rheometer. A 40 mm aluminium cone at 4° was used with an operating gap of 300 µm gap and a solvent trap. For all gels 5 ml of the solution was prepared and added to the plate with a syringe in the operating gap so the gel could form in contact with the cone.

1.6.1 Time sweep experiments: Commenced immediately upon addition and dissolution of the appropriate amount of **GdL** to the gelator solutions. All samples were recorded over 15 h at 20 ^{o}C with a torque of 100 µNm and a frequency of 1 Hz, data was taken every 30 s for a sampling time of 3 s. Some of the time sweeps show unusual abrupt changes; this was due to syneresis and partial drying of samples, which could not be removed experimentally by varying instrumental parameters.

1.6.2 Frequency sweep experiment: Performed immediately after the time sweep at 20 ^{o}C. The torque was kept constant at 100 µNm. Data was obtained for the Frequency sweep from 0.01 Hz to 100 Hz, as a log scale. We have chosen to plot the Frequency sweeps' x axes not as log scales in this paper as the data overlapped too significantly at lower frequencies (which are always noisier) to convey clear differences in the materials' properties.

1.6.3 Stress sweep experiments: Recorded after frequency sweep experiments. All samples were recorded at 20 ^{o}C and at a frequency of 1 Hz.

1.8 pH meter:

The pH meter used was a Mettler Toledo FiveEasy, a two point calibration was performed each morning.

1.9 Mass Spectrometery:

Mass spectrometry data were recorded using electrospray ionization (ESI) in negative mode using a Bruker micrOTOF instrument.

2.0 Synthetic Details and Compound Analysis

2.1 Synthesis of 1,3,5-triformylphloroglucinol (A)

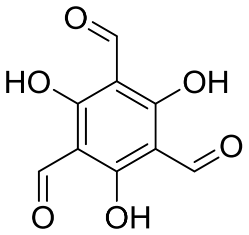

Figure S1. 1,3,5-triformylphloroglucinol (A).

To a mixture of hexamethylenetetramine (22.2 g, 157.5 mmol, 2.2 equiv.) and phloroglucinol (9 g, 71.4 mmol, 1 equiv) trifluoroacetic acid (135 ml) was added, the solution was then heated to 100 °C and stirred under nitrogen for 2.5 h. To the reaction mixture 3 M HCl (300 ml) was slowly added with continued heating at 100 °C and stirred for 1 h. After being allowed to cool to room temperature the reaction mixture was filtered and the filtrate obtained was extracted with dichloromethane (5 × 100 ml) before being dried with $MgSO_4$. Once dry the dichloromethane was removed by rotary evaporation yielding an orange solid. This solid was washed with hot ethanol (3 × 100 ml) to yield a \pale yellow free flowing powder. This powder was dried in the oven overnight to yield the final product ***I***. The synthesis is a scaled up version of the synthesis presented by Chong *et .al* [1].

2.1.1 Yield: 19%, 2.96 g (210.14 gmol^{-1})

2.1.2 ^{1}H NMR(d$_6$-DMSO, J/Hz, δ/ppm): 14.13 (s, 3H, Ar-OH), 10.85 (s, 3H, CHO)

2.1.3 ^{13}C NMR ($CDCl_3$, δ/ppm): 189.07 (-CHO), 178.43 (C-OH), 105.23 (C-aldehyde)

2.1.4 Elemental analysis: Calculated %C = 51.44, %H = 2.88 Found %C = 51.17, %H = 2.92

2.1.5 Melting point: 198-199 °C (literature mp: 198 – 200 °C)[1]

2.2 *Ex Situ* Synthesis of gelators R_1 to R_9 inclusively

To a suspension of A (.250 mg, 1.19 mmol) in ethanol (50 ml) the appropriate amine was added (3.69 mmol, 3.1 molar equivalents) and the resultant suspension was refluxed for 16 hours. Once complete the reflux was cooled to room temperature then cooled to 0 ^{o}C, before the reaction mixture was filtered to obtain a yellow precipitate. The precipitate was washed with hot ethanol (5 × 50 ml) then dried overnight at 80 ^{o}C this produced the desired product at good yields and purity.

Table S1 Reaction details for gelators R_1 to R_9 inclusively

Gelator	Amine	Mass of amine used (mg)	Mass yield (mg)	% yield	Molecular weight of gelator (gmol^{-1})
**R_1**	4-aminobenzoic acid	506	561	83	567.50
**R_2**	5-amino-2-chlorobenzoic acid	633	679	85	670.84
**R_3**	4-amino-2-methoxybenzoic acid	617	618	79	657.58
**R_4**	4-amino-3-hydroxybenzoic acid	565	659	90	615.50
**R_5**	3-amino-4-hydroxybenzoic acid	565	645	88	615.50
**R_6**	3-amino-5-(trifluoromethyl)benzoic acid	757	725	79	771.50
**R_7**	3-amino-2-methoxybenzoic acid	617	571	73	657.58
**R_8**	4-amino-2-methylbenzoic acid	558	638	88	609.58
**R_9**	4-aminophenol	403	512	89	483.47

2.3 Analysis of *ex situ* prepared gelators R_1 to R_9 inclusively

2.3.1 Gelator R_1

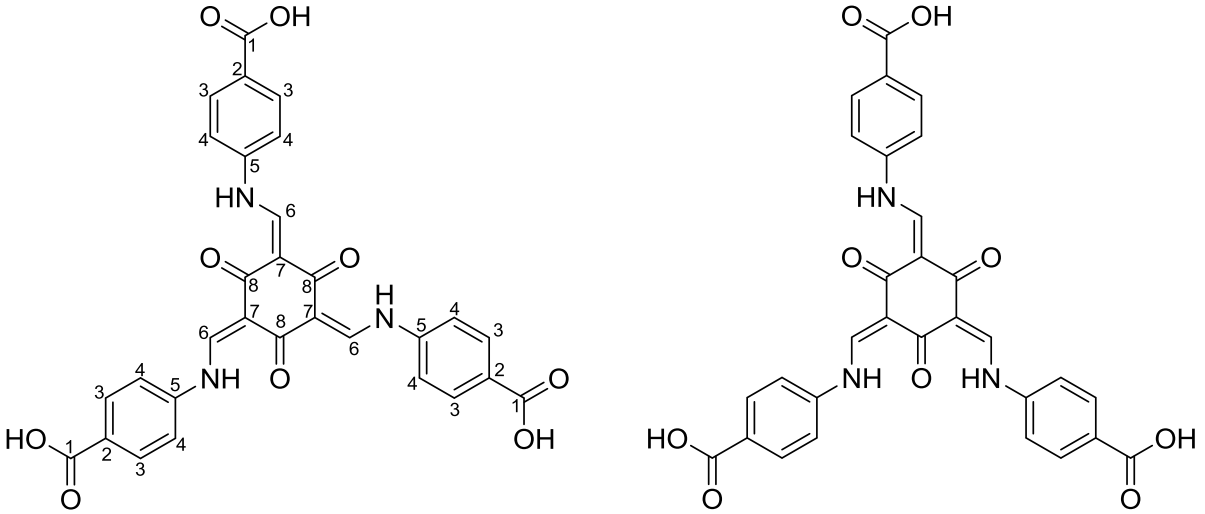

Figure S2. Gelator R_1 showing both conformers C_3 (left) and C_s (right).

2.3.1.1 ^{1}H NMR (d_6-DMSO, J/Hz, δ/ppm): 13.33 (d, J = 12.70, =CNH), 12.86 (dd, J = 11.47, 8.83, =CNH), 8.74 (d, J = 12.76, 3H, HC-N), 7.99 (d, J = 2.41, 6H, Ar-H), 7.58 (dd, J= 16.88, 8.16, 6H, Ar-H).

2.3.1.2 ^{13}C NMR (D_2O, δ/ppm): 187.94, 187.58, 186.93, 175.75, 171.97, 149.75, 130.88, 130.58, 126.21, 116.75, 115.10, 112.05, 111.87, 107.32.

2.3.1.3 IR (cm^{-1}): 3065, 1712, 1623, 1595, 1570, 1455, 1427, 1304, 1229, 1175, 1100, 1034, 995, 847, 764, 686, 649, 635, 578.

2.3.1.4 HRMS: Calculated for $[M-H]^-$ 566.1205, $C_{30}H_{20}N_3O_9$ found 566.1191.

2.3..1.5 Melting point: Compound decomposes upon heating (.>300 °C).

2.3.2 Gelator R_2

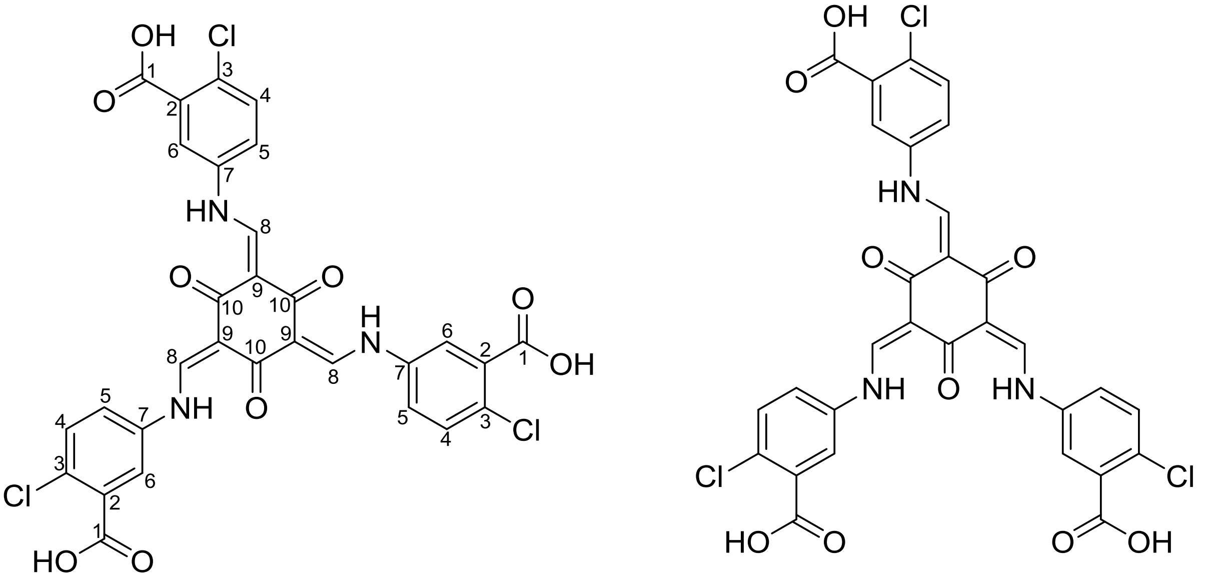

Figure S3. Gelator R_2 showing both conformers C_3 (left) and C_s (right).

2.3.2.1 ^{1}H NMR (d_2-D_2O, J/Hz, δ/ppm): 9.51 (s, 3H, HC-N), 7.29 (s, 6H, Ar-H), 7.02 (s, 3H, Ar-H).

2.3.2.2 ^{13}C NMR (D_2O, δ/ppm): 187.93, 187.42, 186.94, 175.88, 173.73, 147.88, 145.00, 139.76, 139.45, 138.06, 135.85, 131.22, 130.16, 126.68, 118.47, 117.35, 116.17, 114.87, 112.08, 106.01.

2.3.2.3 IR (cm^{-1}): 2987, 2589, 1725, 1605, 1568, 1548, 1444, 1409, 1340, 1294, 1270, 1228, 1121, 1037, 985, 890, 816, 763, 695, 665.

2.3.1.4 HRMS: Calculated for $[M-H]^-$ 668.0036, $C_{30}H_{17}Cl_3N_3O_9$ found 668.1007.

2.3..1.5 Melting point: Compound decomposes upon heating (.>300 $^{\circ}$C).

2.3.3 Gelator R_3

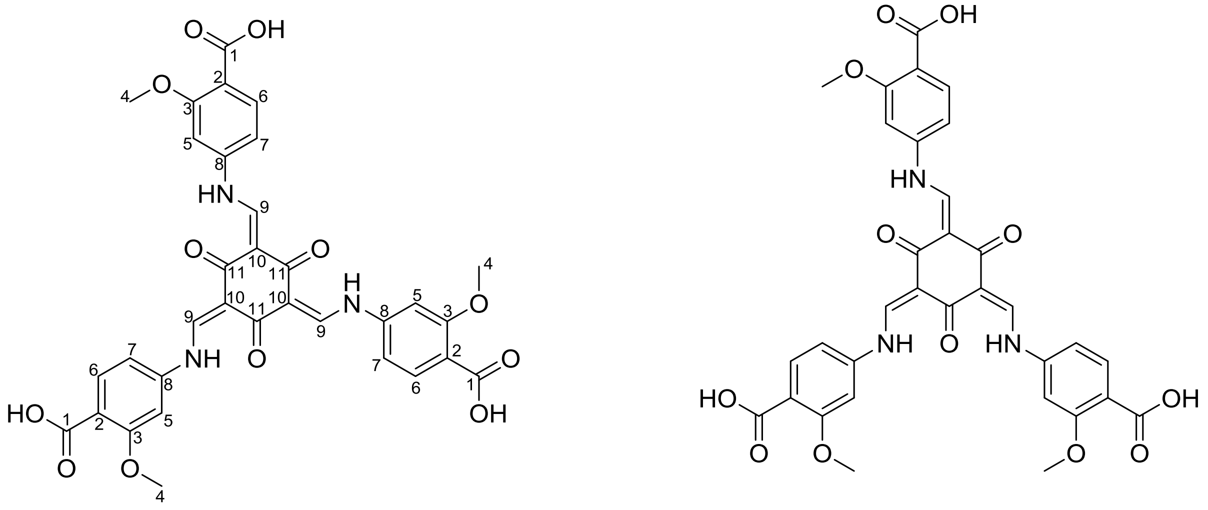

Figure S4. Gelator R_3 showing both conformers C_3 (left) and C_s (right).

2.3.3.1 ^{1}H NMR (d_6-DMSO, J/Hz, δ/ppm): 13.92 (d, J = 13.00, =CNH), 13.29 (dd, J = 24.67,13.41, CNH), 8.80 (m, 3H, HC-N), (td, J = 7.71,3.75, 3H, Ar-H), 7.22 (m, 6H, Ar-H), 3.90 (d, J = 3.63, 9H, OCH_3).

2.3.3.2 ^{13}C NMR (D_2O, δ/ppm): 187.44, 187.0, 175.87, 158.56, 149.82, 131.56, 117.71, 112.12, 107.33, 99.50, 55.22.

2.3.3.3 IR (cm^{-1}): 3010, 1725, 1627, 1565, 1468, 1446, 1296, 1216, 1153, 1075, 1024, 948, 871, 796, 763, 645, 560.

2.3.3.4 HRMS: Calculated for $[M-H]^-$ 656.1522, $C_{33}H_{26}N_3O_{12}$ found 668.1581.

2.3.3.5 Melting point: Compound decomposes upon heating (.>300 ^oC).

2.3.4 Gelator R_4

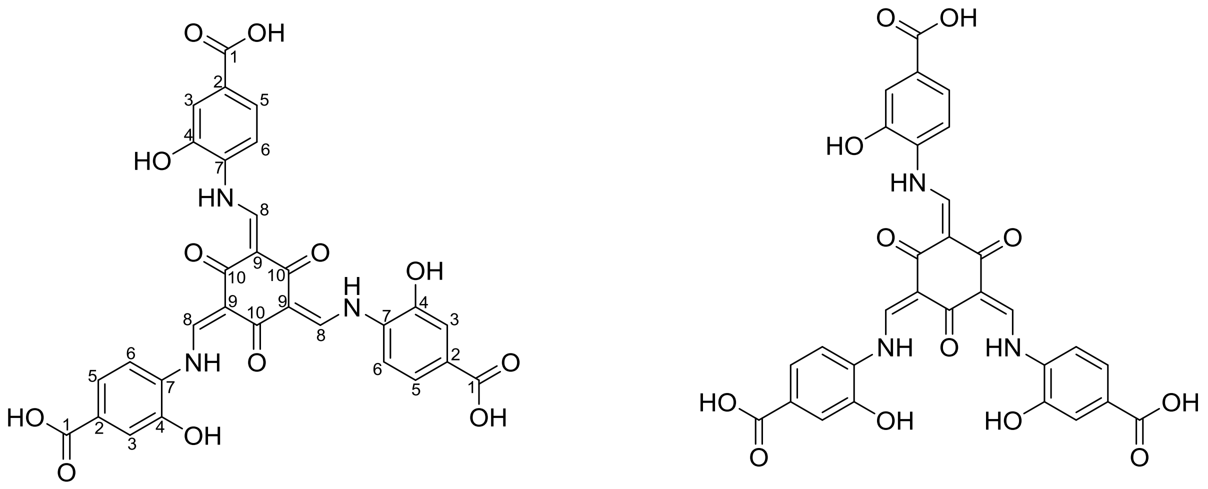

Figure S5. Gelator R_4 showing both conformers C_3 (left) and C_s (right).

2.3.4.1 ^{1}H NMR (d_6-DMSO, J/Hz, δ/ppm): 13.99 (d, J = 13.29, =CNH), 13.50 (dd, J = 28.00, 13.31, =CNH), 12.81 (s, 3H-OH), 8.78 (m, 3H, HC-N), 7.74 (dt, J = 8.57,4.52, 3H, Ar-H), 7.48 (t, J = 7.49, 6H, Ar-H)

2.3.4.2 ^{13}C NMR (D_2O, δ/ppm): 188.04, 187.4, 187.13, 176.63, 176.02, 156.77, 154.26, 147.19, 140.64, 133.65, 132.50, 127.79, 119.50, 117.67, 116.45, 115.46, 114.70, 113.79, 111.96, 106.41.

2.3.4.3 IR (cm^{-1}): 3394, 3263, 1662, 1575, 1530, 1429, 1353, 1277, 1196, 1142, 1092, 988, 949, 884, 841, 816, 758, 721, 672, 635, 570.

2.3.4.4 HRMS: Calculated for $[M-H]^-$ 614.1052, $C_{30}H_{20}N_3O_{12}$ found 614.1233.

2.3.4.5 Melting point: Compound decomposes upon heating (.>300 °C).

2.3.5 Gelator R_5

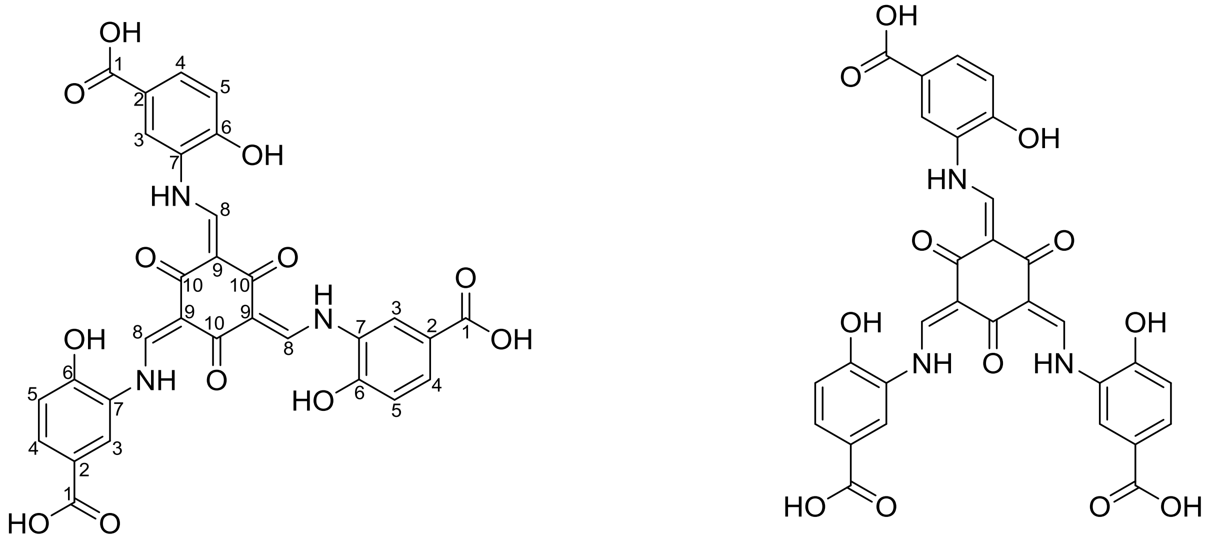

Figure S6. Gelator R_5 showing both conformers C_3 (left) and C_s (right).

2.3.5.1 ^{1}H NMR (d_6-DMSO, J/Hz, δ/ppm): 13.29 (dd, J = 18.92, 13.56, =CNH), 12.83 (dd, J = 13.59, 8.14, =CNH), 11.36 (s, 3H, –OH), 8.74 (dd, J = 13.37,2.88, 3H, HC-N), 8.01 (d, J = 16.78, 3H Ar-H), 7.69 (d, J = 8.36, 3H, Ar-H), 7.08 (m, 3H, Ar-H).

2.3.5.2 ^{13}C NMR (D_2O, δ/ppm): 176.51, 175.81, 162.17, 148.35, 135.76, 129.25, 128.96, 128.61, 123.52, 121.28, 118.70, 117.29, 116.59, 105.97.

2.3.5.3 IR (cm^{-1}): 3076, 1693, 1580, 1450, 1320, 1250, 1121, 1093, 999, 941, 820, 761, 614, 562.

2.3.5.4 HRMS: Calculated for $[M\text{-}H]^-$ 614.1052, $C_{30}H_{20}N_3O_{12}$ found 614.1759.

2.3.5.5 Melting point: Compound decomposes upon heating (.>300 °C).

2.3.6 Gelator R_6

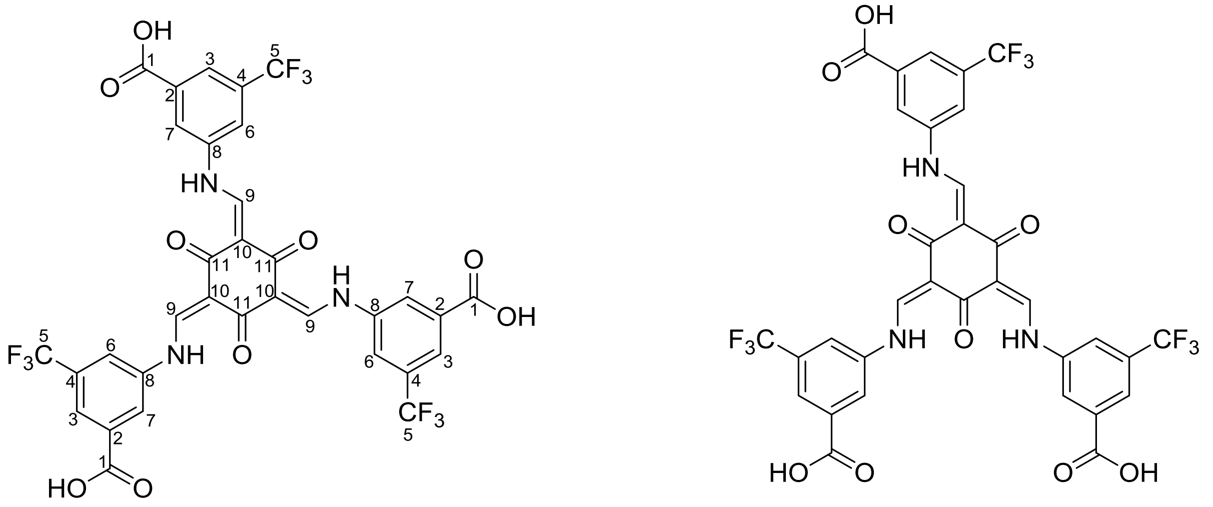

Figure S7. Gelator R_6 showing both conformers C_3 (left) and C_s (right).

2.3.6.1 ^{1}H NMR (d_2-D_2O, J/Hz, δ/ppm): 9.45 (s, 3H, HC-N), 7.36 (s, 3H, Ar-H), 7.11 (s, 6H, Ar-H).

2.3.6.2 ^{13}C NMR (D_2O, δ/ppm): 187.22, 173.94, 147.12, 138.25, 131.19, 130.81, 130.28, 129.93, 129.86, 129.35, 125.75, 122.13, 119.39, 115.69, 114.40, 111.99.

2.3.6.3 IR (cm^{-1}): 3079, 2577, 1715, 1621, 1586, 1471, 1355, 1274, 1172, 1124, 1042, 946, 870, 829, 768, 734, 675, 607, 571, 562.

2.3.6.4 HRMS: Calculated for $[M-H]^-$ 770.0827, $C_{33}H_{17}F_9N_3O_9$ found 770.0495.

2.3.6.5 Melting point: Compound decomposes upon heating (.>300 °C).

2.3.7 Gelator R_7

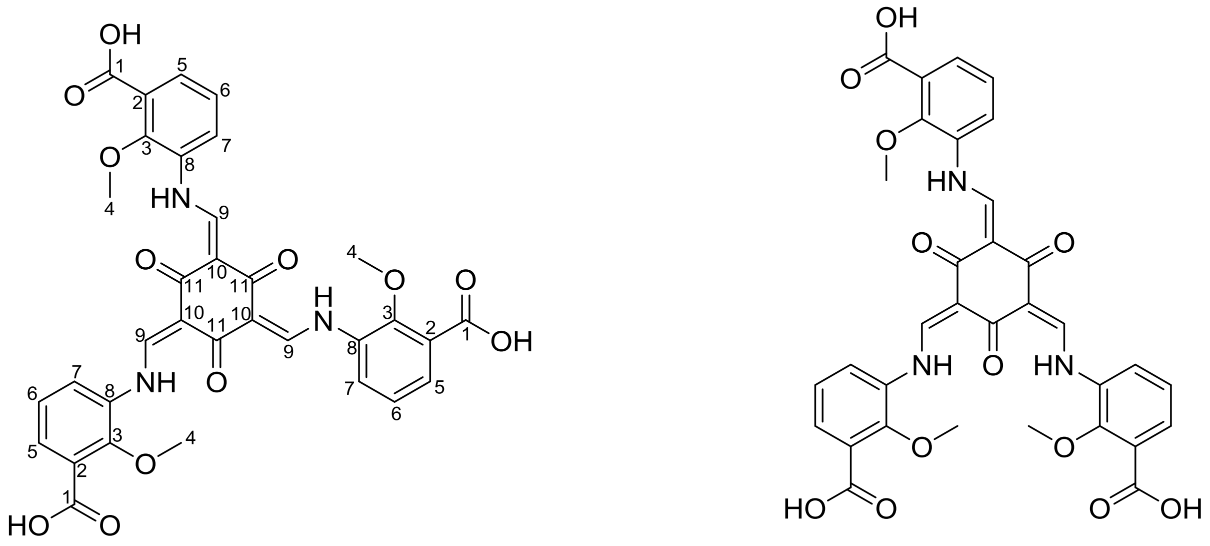

Figure S8. Gelator R_7 showing both conformers C_3 (left) and C_s (right).

2.3.7.1 ^{1}H NMR (d_2-D_2O, J/Hz, δ/ppm): 9.57 (s, 3H, HC-N), 7.40 (s, 3H, Ar-H), 7.04 (s, 3H, Ar-H), 6.70 (s, 3H, Ar-H).

2.3.7.2 ^{13}C NMR (D_2O, δ/ppm): 188.34, 187.91, 187.09, 178.68, 177.46, 148.41, 147.79, 147.01, 139.26, 138.45, 137.76, 137.24, 137.07, 135.84, 129.97, 129.26, 128.47, 118.91, 117.74, 114.10, 113.01, 111.83, 106.87, 106.18, 20.17, 19.82.

2.3.7.3 IR (cm^{-1}): 2973, 2613, 1698, 1623, 1563, 1448, 1344, 1284, 1217, 1143, 1039, 984, 821, 770, 751, 688, 648, 567.

2.3.7.4 HRMS: Calculated for $[M-H]^-$ 656.1522, $C_{33}H_{26}N_3O_{12}$ found 668.1085.

2.3.7.5 Melting point: Compound decomposes upon heating (.>300 ºC).

2.3.8 Gelator R_8

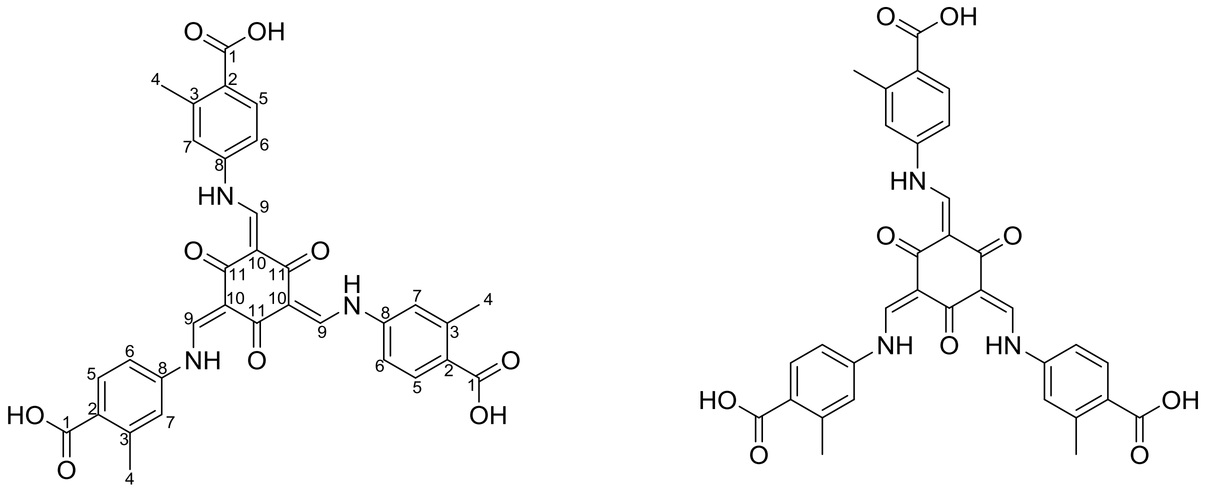

Figure S9. Gelator R_8 showing both conformers C_3 (left) and C_s (right).

2.3.8.1 ^{1}H NMR (d_2-D_2O, J/Hz, δ/ppm): 9.43 (d, J = 11.43, 3H, HC-N), 7.32 (s, 3H, Ar-H), 7.04 (s, 3H, Ar-H), 6.61 (s, 3H, Ar-H).

2.3.8.2 ^{13}C NMR (D_2O, δ/ppm): 187.93, 187.47, 186.97, 176.47, 173.75, 146.46, 144.01, 139.06, 133.37, 131.45, 130.40, 126.09, 124.53, 124.23, 118.60, 117.84, 114.45, 112.02, 106.25, 61.81, 60.92.

2.3.8.3 IR (cm^{-1}): 3079, 1705, 1623, 1566, 1454, 1420, 1221, 1170, 1113, 1091, 1034, 985, 846, 764, 688, 634.

2.3.8.4 HRMS: Calculated for $[M-H]^-$ 608.1675, $C_{33}H_{26}N_3O_9$ found 608.1752.

2.3.8.5 Melting point: Compound decomposes upon heating (.>300 ^{o}C).

2.3.9 Gelator R_9

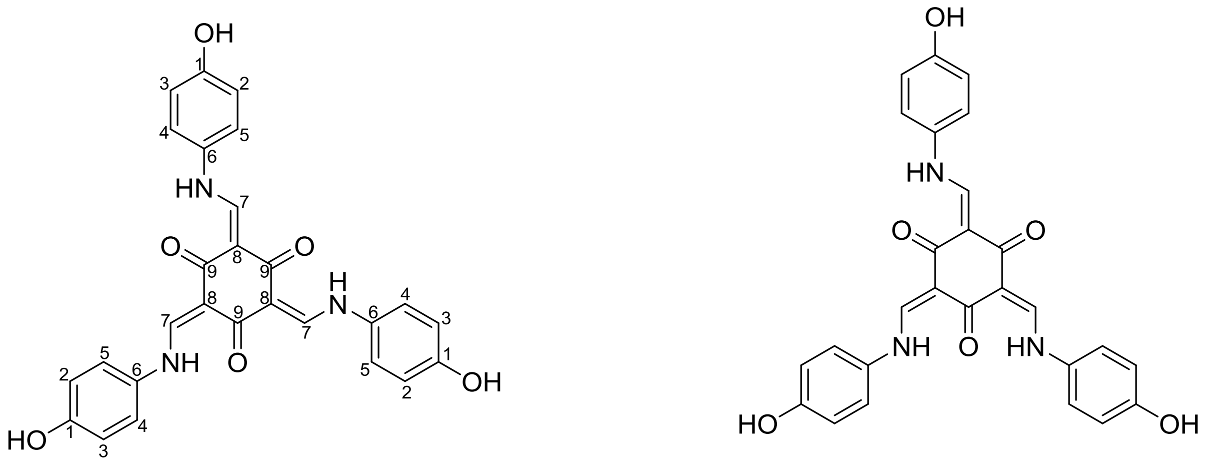

Figure S10. Gelator R_9 showing both conformers C_3 (left) and C_s (right).

2.3.9.1 ^{1}H NMR (d_6-DMSO, J/Hz, δ/ppm): 13.36 (t, J = 13.11, =CNH), 12.77 (dd, J = 13.50, 4.15, =CNH), 9.61 (s, 3H, Ar-OH), 8.53 (m, 3H, HC-N), 7.28 (dd, J = 7.71, 5.45, 6H, Ar-H), 6.84 (d, J = 8.39, 6H, Ar-H).

2.3.9.2 ^{13}C NMR (D_2O, δ/ppm): 187.78, 185.80, 184.00, 165.22, 159.52, 148.51, 147.89, 133.32, 126.51, 126.17, 119.36, 118.85, 111.92, 104.85.

2.3.9.3 IR (cm^{-1}): 3079, 1705, 1623, 1566, 1454, 1420, 1221, 1170, 1113, 1091, 1034, 985, 846, 764, 688, 634.

2.3.9.4 HRMS: Calculated for $[M-H]^-$ 482.1358, $C_{27}H_{20}N_3O_6$ found 482.1731.

2.3.9.5 Melting point: Compound decomposes upon heating (.>300 ^{o}C).

2.4 Synthesis of compound R_{10}

To a suspension of A (.250 mg, 1.19 mmol) in ethanol (50 ml) 6-aminohexanoic acid was added (3.69 mmol, 3.1 molar equivalents) and the resulting solution was refluxed for 16 hours. Once complete the reflux was cooled to room temperature and rotary evaporation was used to remove all the ethanol resulting in a dark orange oil. After a week at 5 °C the oil had solidified to give a dark orange solid. The solid ground to a fine powder and washed with hot ethanol (5 × 50 ml) then dried overnight at 80 °C this produced the desired product at good yields (71 %) and purity.

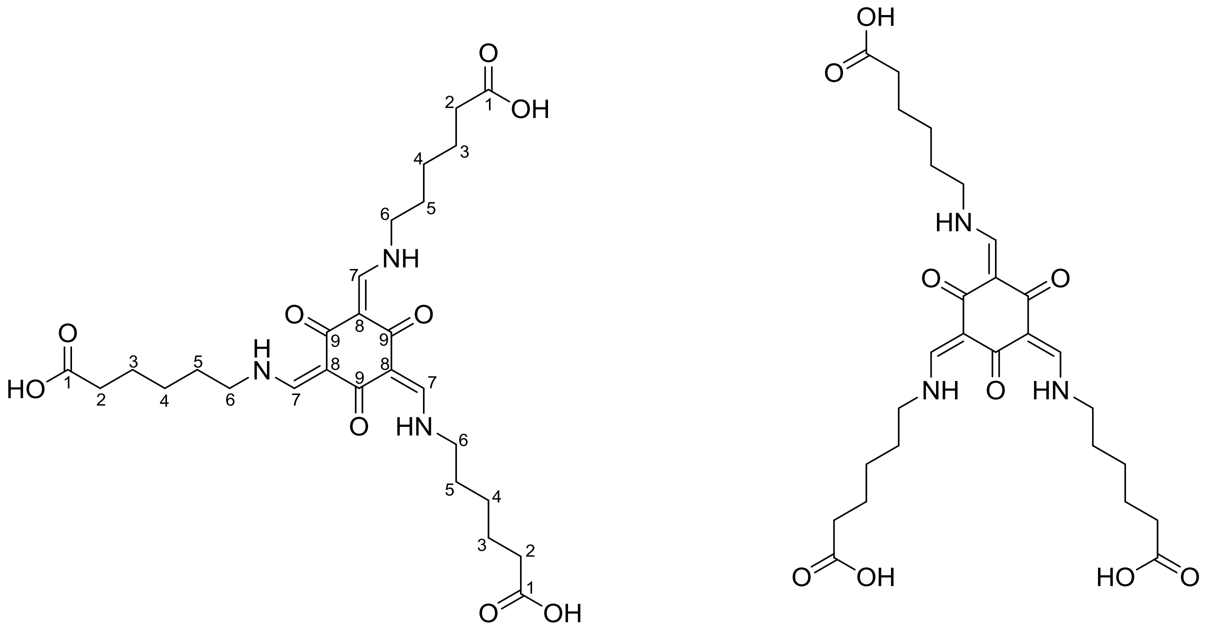

Figure S11. Compound R_{11} showing both conformers *C_3* (left) and *C_s* (right).

2.5.1 ^{1}H NMR (d_6-DMSO, J/Hz, δ/ppm): 11.31 (m, =CNH), 10.87 (m, =CNH), 8.06 (m, 3H, HC-N), 3.40 (m, 6H, N-CH_2-), 2.18 (t, J = 6.96, 6H, -CH_2-), 1.52 (dt, J = 15.06, 7.47, 12H, -CH_2-), 1.30 (m, 6H, -CH_2-).

2.5.2 ^{13}C NMR (d_6-DMSO, δ/ppm): 187.19, 184.26, 181.25, 175.20, 157.78, 157.31, 157.16, 156.55, 104.26, 104.07, 49.62, 49.42, 34.47, 30.49, 27.36, 26.07, 24.75.

2.5.3 IR (cm^{-1}): 2932, 2860, 1716, 1601, 1525, 1450, 1306, 1160, 1105, 1074, 1021, 832, 776, 732, 655.

2.5.4 HRMS: Calculated for $[M-H]^-$ 548.2608 $C_{27}H_{38}N_3O_9$ found 548.2588.

2.5.5 Melting point: 128 – 132 °C.

2.5 Chemical Analysis notes

Ligands R_2, R_6, R_7 and R_8 prooved too insoluble in any solvent to obtain a ^1H spectra of the pure compounds. This resulted in proton data experiments having to be run in D_2O with the addition of NaOH in order to solublise the ligand. To obtain chemical information for the ligands the spectra where recorded between 5 and 15 ppm in order to remove the H_2O signal which would have otherwise suppressed the signals for the ligand ^1H nuclei. Due to deportonation and exchange, the –NH- and –COOH signals were lost.

All ^{13}C NMR spectra with the exception of R_{10} were recorded in the manner described above. Due to the two distinct conformations of the ligands and the overlapping of the subsequent signals exact assignment of signals proved impossible.

3.0 Methods for hydrogel Preparation.

Nine hydrogels could be prepared with two preparation methods attributed to each individual hydrogel. The *'ex situ'* preparation method describes the use of the previously prepared compounds R_1 to R_9 to form the corresponding hydrogel. The '*in situ*' preparation method relates to the mixing and subsequent reaction of the amine and aldehyde component of the gelator in the volume of water that is to be gelled.

3.1 Setting gel using *In situ* gel preparation method (gels R_1 to R_8 inclusively):

This procedure describes the method used in a typical in situ setting of the hydrogels using a solution of 1,3,5-triformylglucinol and the appropriate aminobenzoic acid.

1,3,5-triformylglucinol (0.082g, 0.39 mmol) was suspended in water (5 ml), to this suspension sodium hydroxide (0.05 g, 1.25 mmol) was added and sonication and gentle heating was used to produce a clear pale yellow solution. To a separate portion of water (5 ml) the appropriate aminobenzoic acid was added (1.17 mmol) along with sodium hydroxide (0.016 g, 0.4 mmol) to ensure the complete dissolution of the aminobenzoic acid. These two aqueous solutions were then mixed together vigorously shaken before being allowed to react at room temperature for 4 hours. After this reaction period glucono *delta*-lactone (**GdL**) (0.08 g, 0.45 mmol) was added and the solution was shaken until the **GdL** had completely dissolved, this resulted in the formation of the gel after approximately 2 hours.

3.2 *In situ* R9 gel formation:

This procedure describes the method used in a typical in situ setting of the hydrogel R_9 using a solution of 1,3,5-triformylglucinol and the 4-aminophenol.

1,3,5-triformylglucinol (0.082g, 0.39 mmol) was suspended in water (5 ml), to this suspension sodium hydroxide (0.05 g, 1.25 mmol) was added and sonication and gentle heating was used to produce a clear pale yellow solution. To a separate portion of water (5 ml) the 4-aminophenol was added (0.13 g, 1.17 mmol) along with sodium hydroxide (0.016 g, 0.4 mmol) to ensure the complete dissolution of the 4-aminophenol. These two aqueous solutions were then mixed together vigorously shaken before being allowed to react at room temperature for 4 hours. After this reaction period conc. Hydrochloric acid (0.8 ml) was rapidly added to the solution instantly producing a bright orange gel.

3.3 Setting gel using the *ex situ* prepared compound (gels R_1 to R_8 inclusively):

This procedure describes the method used in a typical setting of the hydrogel at 2 wt% using the previously prepared synthesised compound.
The gel ligand (0.1 g) was suspended in water, to this suspension sodium hydroxide was added (0.05 g, 1.25 mmol). Sonication and gentle heating was used to ensure the ligand had completely dissolved giving a solution with a pH of 9 – 10. To this solution glucono delta-lactone (**GdL**) (0.08 g, 0.45 mmol) was added and the solution was shaken until the **GdL** had completely dissolved, this resulted in the formation of the gel after approximately 2 hours.

3.4 Setting gel R9 using the *ex situ* prepared compound:

This procedure describes the method used in a typical setting of the hydrogel R_9 at 2 wt% using the previously prepared synthesised compound R_9.

The synthesised compound R9 (0.1 g, 0.21 mmol) was suspended in water (5 ml), to this suspension sodium hydroxide was added (0.05 g, 1.25 mmol). Sonication and gentle heating was used to ensure the ligand had completely dissolved giving a solution with a pH of 9 – 10. To this solution conc. Hydrochloric acid (0.4 ml) was rapidly added instantly producing a bright orange gel.

3.5 Critical gel concentration (CGC):

Critical gel concentration was determined using the inverted vial method in 2 ml sample vials using 0.5 ml of water. This method can often give lower CGC values than are determined using a rheometer due to the low volume of solution and the high surface tension within the vials.

4.0 Morphology determination of single amine Gels

The morphology of the gels could be determined using SEM images. The gels were prepared by both the *ex situ* and *in situ* in vials before being mounted on carbon covered SEM stubs. The gels were then dried under vacuum for 72 hours before being gold coated.

4.1 Gel R_1

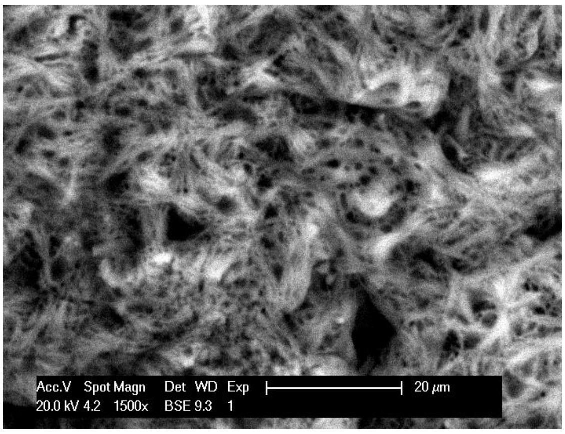

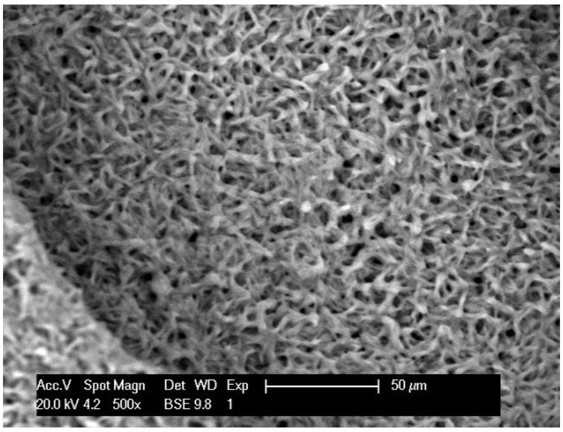

Figure S12. SEM images of gel R_1 *ex situ* preparation method (left) *in situ* preparation method (right).

4.2 Gel R_2

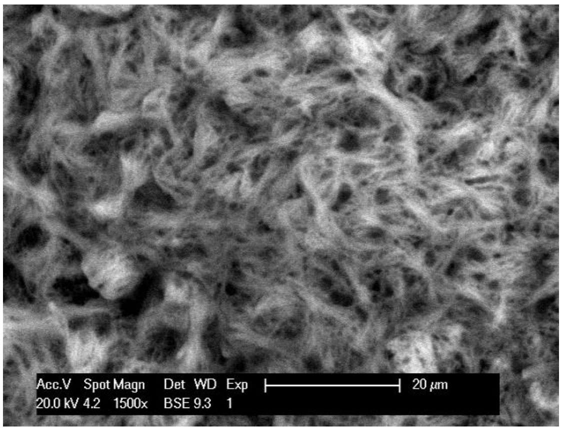

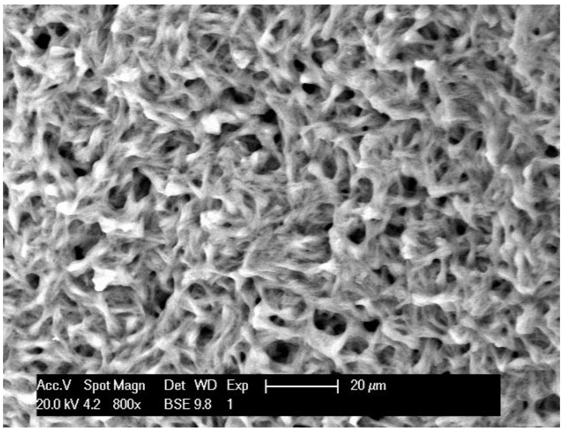

Figure S13. SEM images of gel R_2 *ex situ* preparation method (left) *in situ* preparation method (right).

4.3 Gel R_3

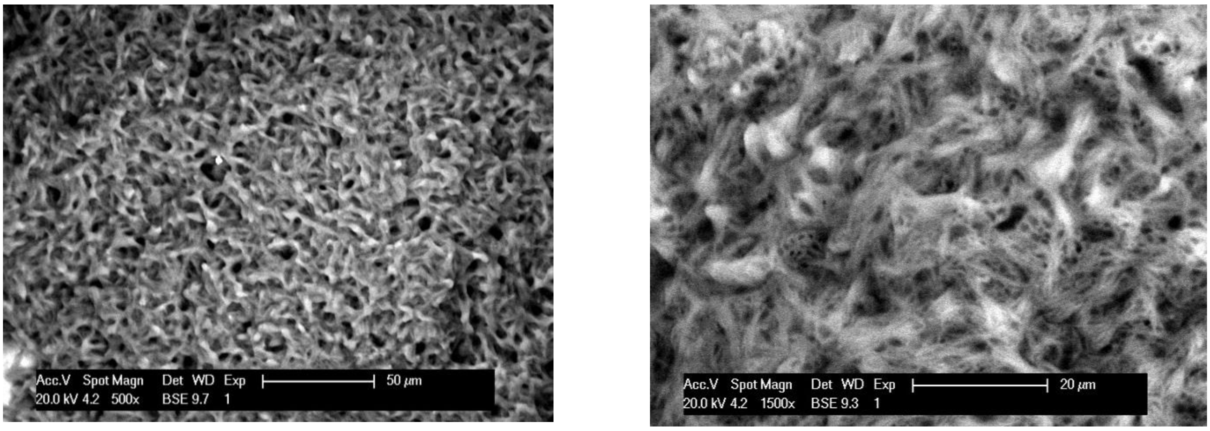

Figure S14. SEM images of gel R_3 *ex situ* preparation method (left) *in situ* preparation method (right).

4.4 Gel R_4

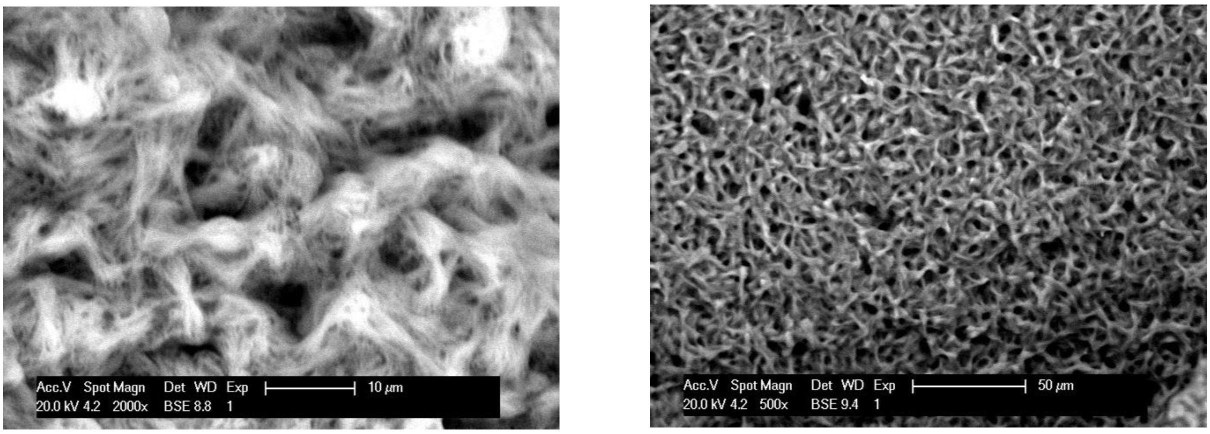

Figure S15. SEM images of gel R_4 *ex situ* preparation method (left) *in situ* preparation method (right).

4.5 Gel R_5

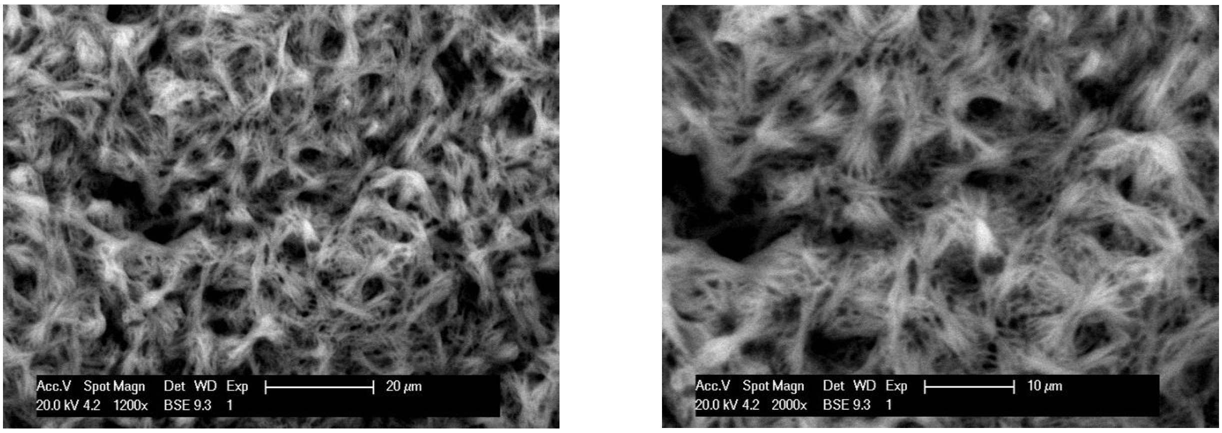

Figure S16. SEM images of gel R_5 *ex situ* preparation method (left) *in situ* preparation method (right).

4.6 Gel R_6

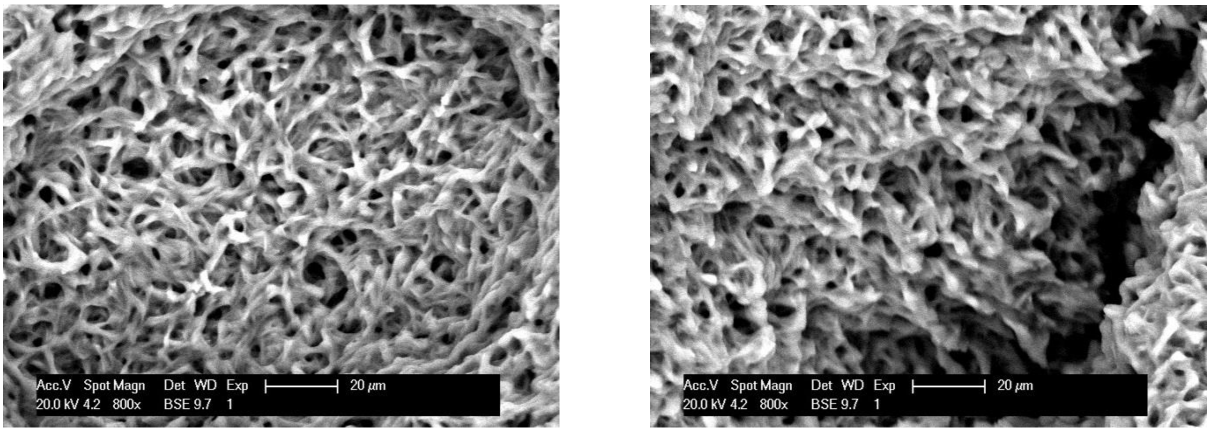

Figure S17. SEM images of gel R_6 *ex situ* preparation method (left) *in situ* preparation method (right).

4.7 Gel R_7

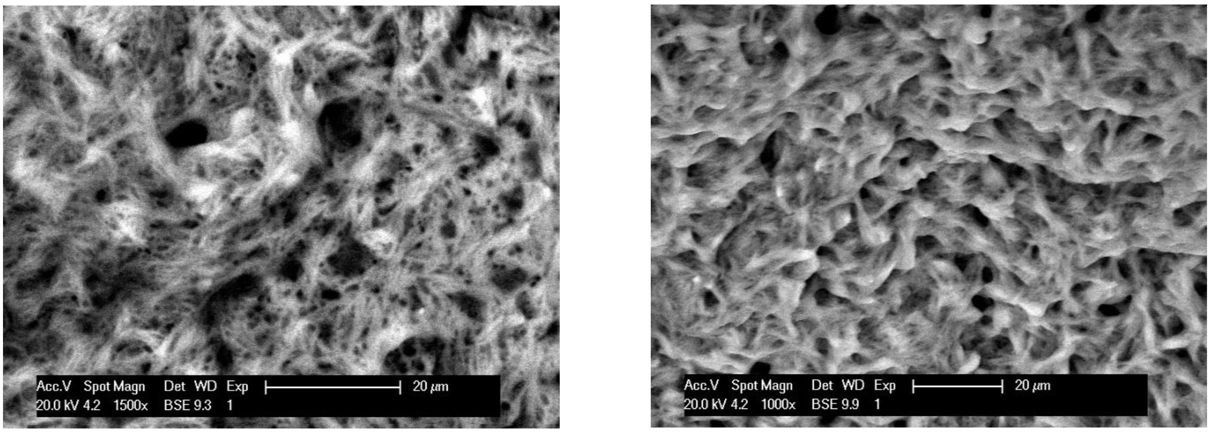

Figure S18. SEM images of gel R_7 *ex situ* preparation method (left) *in situ* preparation method (right).

4.8 Gel R_8

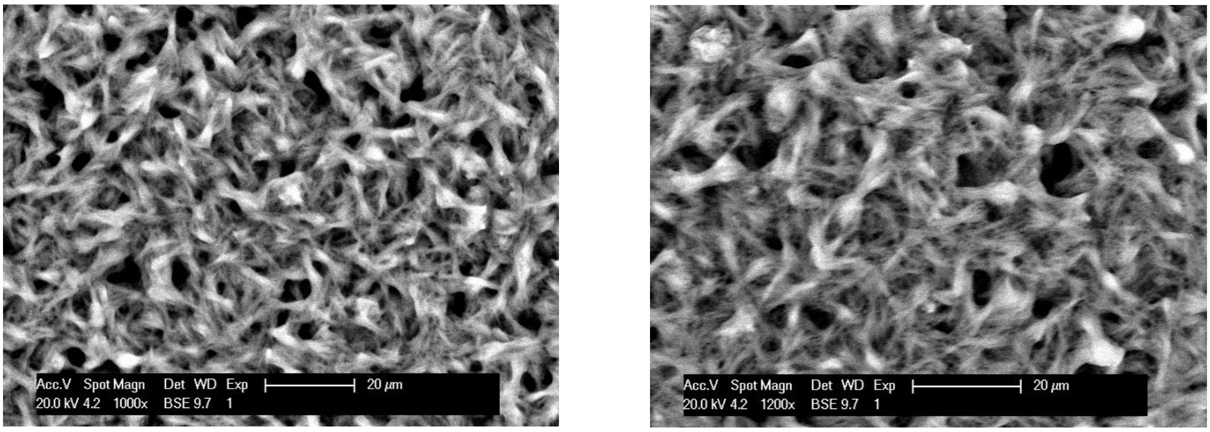

Figure S19 SEM images of gel R_8 *ex situ* preparation method (left) *in situ* preparation method (right).

4.9 Gel R_9

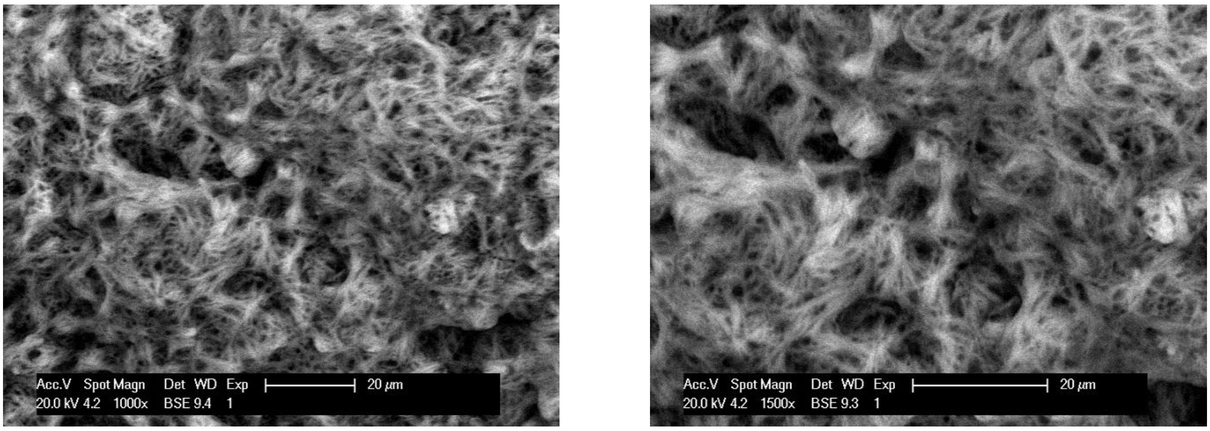

Figure S20. SEM images of gel R_9 *ex situ* preparation method (left) *in situ* preparation method (right).

5.0 Rheological studies of single amine gels

5.1 Rheological characterisation of *in situ* and *ex situ* prepared gel R_1

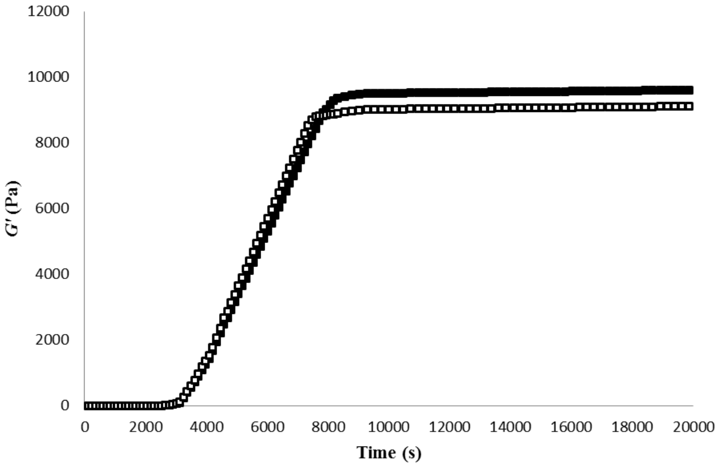

Figure S21. Time sweep rheometry of gel R_1 *ex situ* preparation method (empty □) and *in situ* preparation method (filled □).

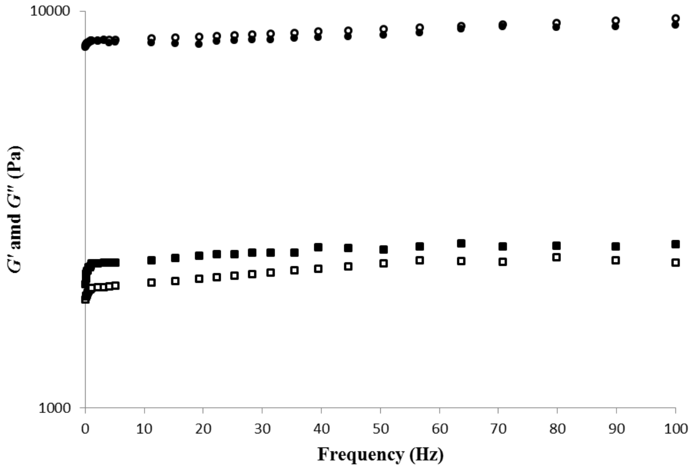

Figure S22. Frequency sweep rheometry of gel R_1 showing *ex situ* preparation method (G' = empty ○, G'' = empty □) and the *in situ* preparation method (G' = filled ○, G'' = filled □).

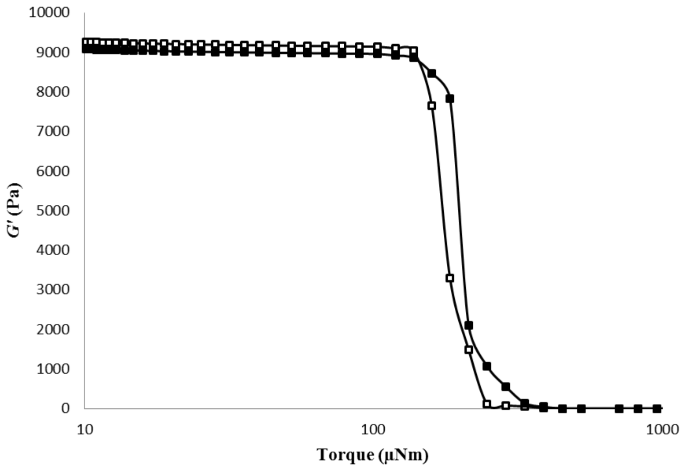

Figure S23. Stess sweep rheometry of gel R_1 showing *ex situ* preparation method (empty □) and *in situ* preparation method (filled □) (line has been added to guide the eye).

5.2 Rheological characterisation of *in situ* and *ex situ* prepared gel R_2

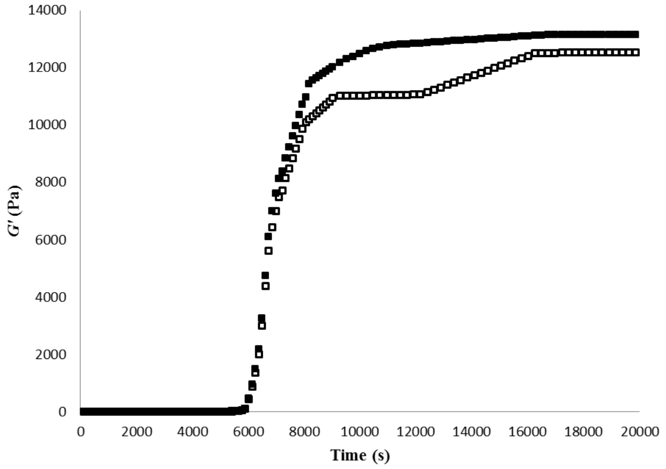

Figure S24. Time sweep rheometry of gel R_2 *ex situ* preparation method (empty □) and *in situ* preparation method (filled □).

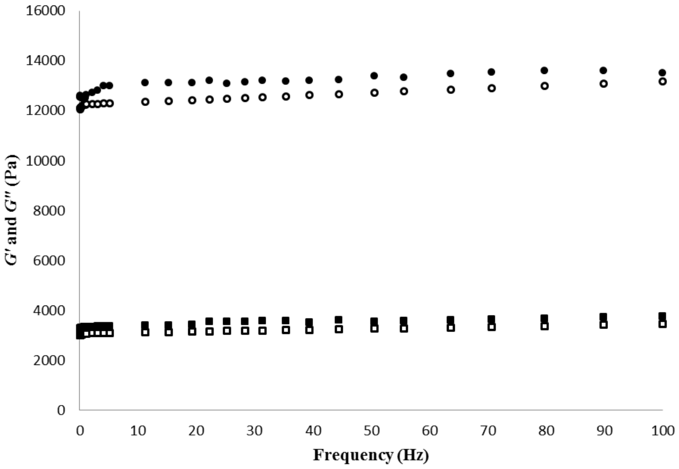

Figure S25. Frequency sweep rheometry of gel R_1 showing *ex situ* preparation method (*G′* = empty ○, *G″* = empty □) and the *in situ* preparation method (*G′* = filled ○, *G″* = filled □).

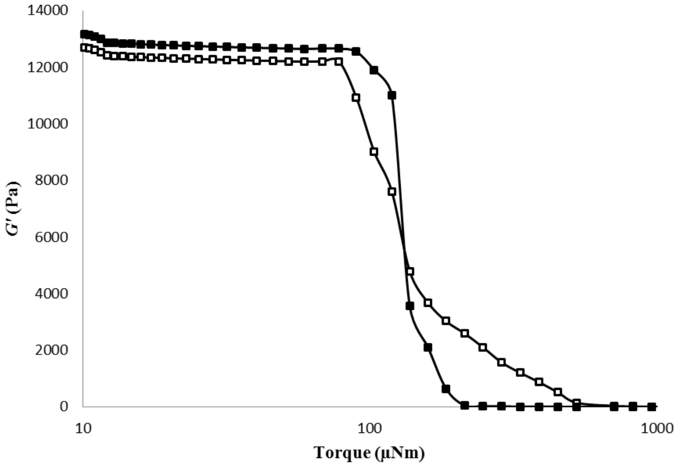

Figure S26. Stess sweep rheometry of gel R_2 showing *ex situ* preparation method (empty □) and *in situ* preparation method (filled □) (line has been added to guide the eye).

5.3 Rheological characterisation of *in situ* and *ex situ* prepared gel R_3

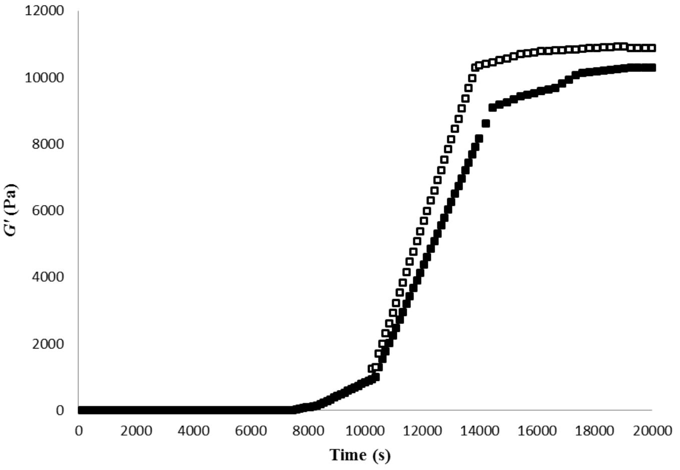

Figure S27. Time sweep rheometry of gel R_3 *ex situ* preparation method (empty □) and *in situ* preparation method (filled □).

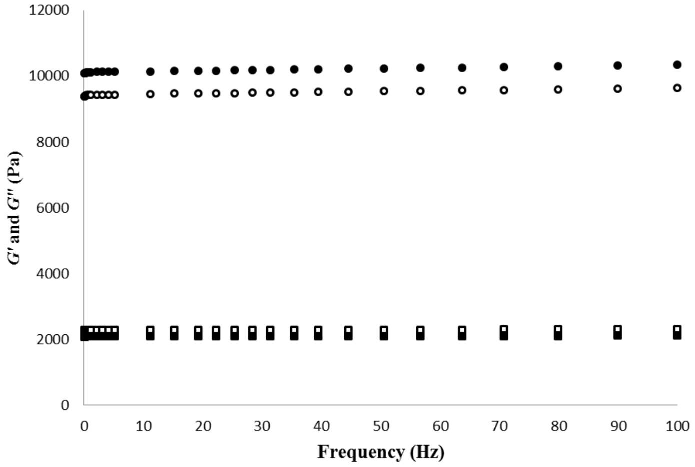

Figure S28. Frequency sweep rheometry of gel R_3 showing *ex situ* preparation method (G' = empty ○, G'' = empty □) and the *in situ* preparation method (G' = filled ○, G'' = filled □).

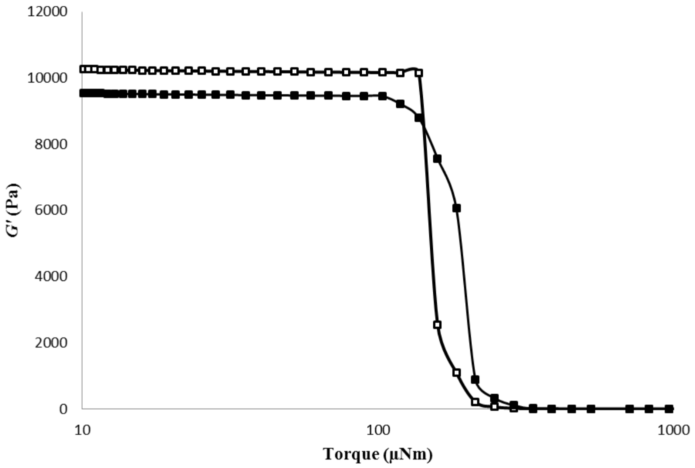

Figure S29. Stess sweep rheometry of gel R_3 showing *ex situ* preparation method (empty □) and *in situ* preparation method (filled □) (line has been added to guide the eye).

5.4 Rheological characterisation of *in situ* and *ex situ* prepared gel R_4

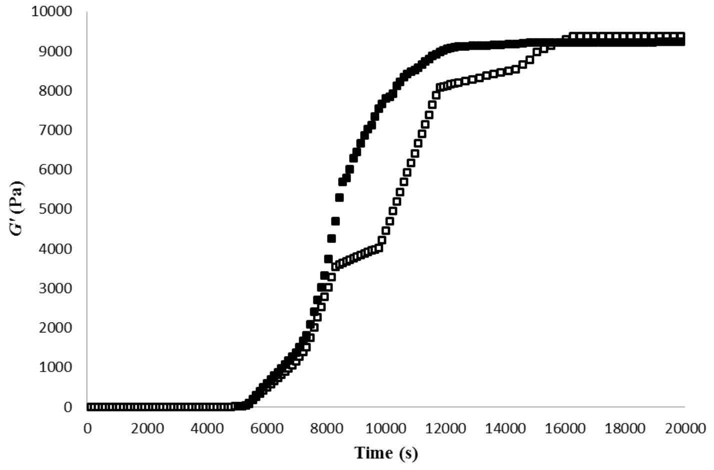

Figure S30. Time sweep rheometry of gel R_4 *ex situ* preparation method (empty □) and *in situ* preparation method (filled □).

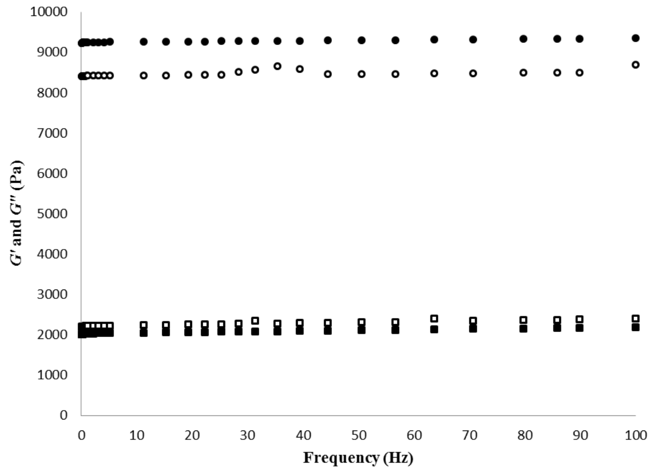

Figure S31. Frequency sweep rheometry of gel R_4 showing *ex situ* preparation method (G' = empty ○, G'' = empty □) and the *in situ* preparation method (G' = filled ○, G'' = filled □).

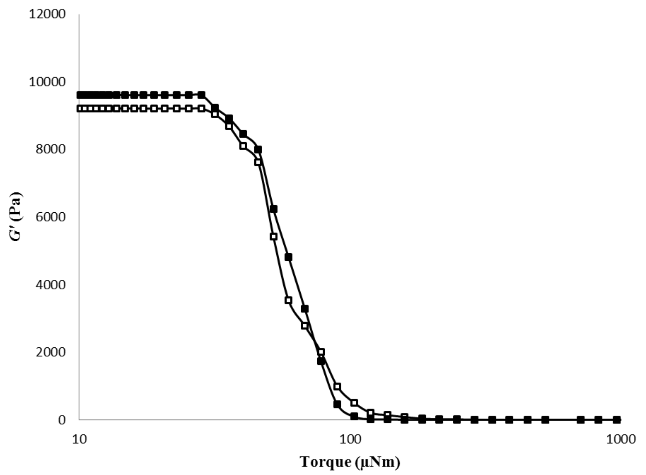

Figure S32. Stess sweep rheometry of gel R_4 showing *ex situ* preparation method (empty □) and *in situ* preparation method (filled □) (line has been added to guide the eye).

5.5 Rheological characterisation of *in situ* and *ex situ* prepared gel R_5

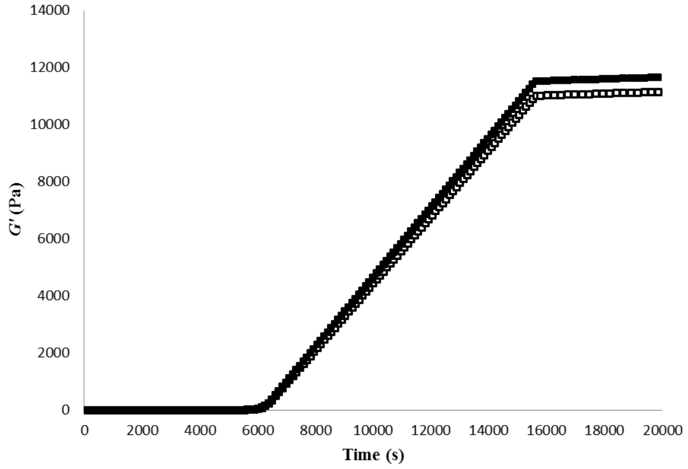

Figure S33. Time sweep rheometry of gel R_5 *ex situ* preparation method (empty □) and *in situ* preparation method (filled □).

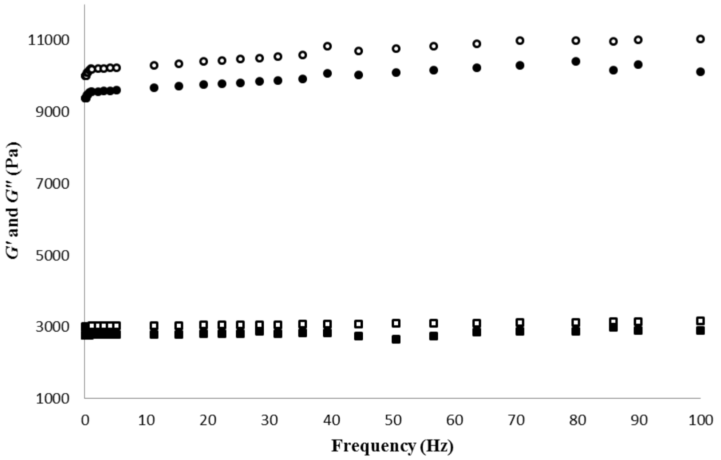

Figure S34. Frequency sweep rheometry of gel R_5 showing *ex situ* preparation method (G' = empty ○, G'' = empty □) and the *in situ* preparation method (G' = filled ○, G'' = filled □).

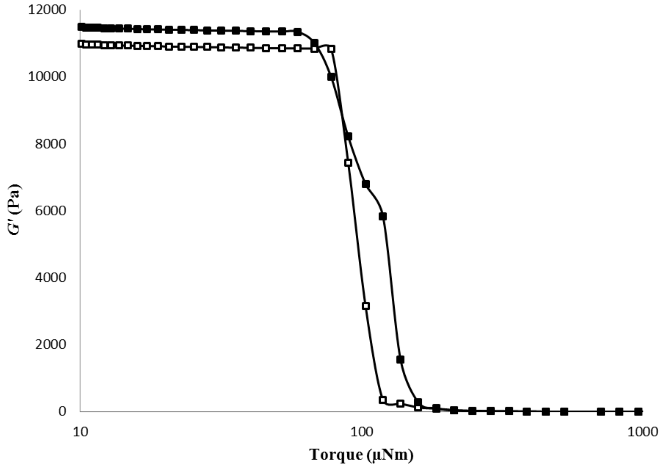

Figure S35. Stess sweep rheometry of gel R_5 showing *ex situ* preparation method (empty □) and *in situ* preparation method (filled □) (line has been added to guide the eye).

5.6 Rheological characterisation of *in situ* and *ex situ* prepared gel R_6

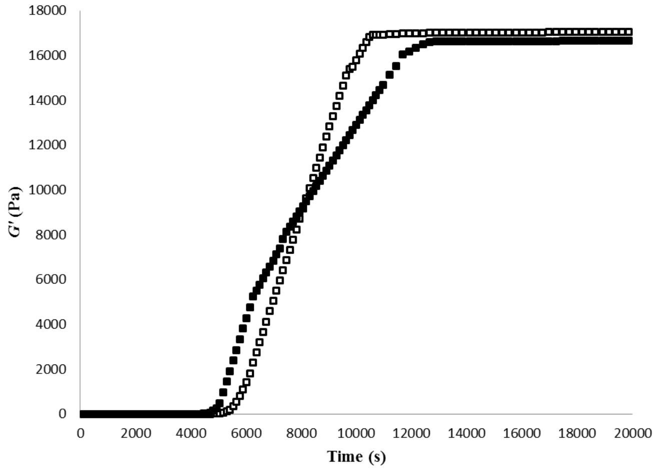

Figure S36. Time sweep rheometry of gel R_6 *ex situ* preparation method (empty □) and *in situ* preparation method (filled □).

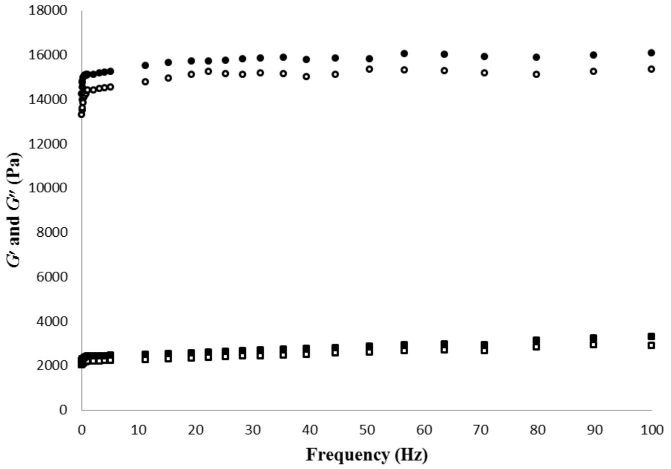

Figure S37. Frequency sweep rheometry of gel R_6 showing *ex situ* preparation method (*G′* = empty ○, *G″* = empty □) and the *in situ* preparation method (*G′* = filled ○, *G″* = filled □).

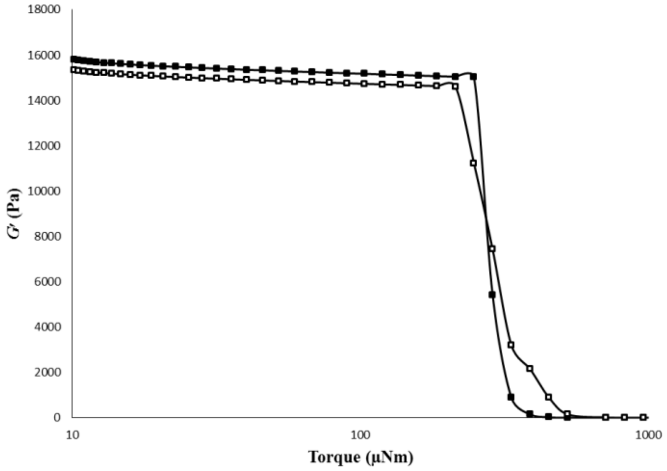

Figure S38 Stess sweep rheometry of gel R_6 showing *ex situ* preparation method (empty □) and *in situ* preparation method (filled □) (line has been added to guide the eye).

5.7 Rheological characterisation of *in situ* and *ex situ* prepared gel R_7

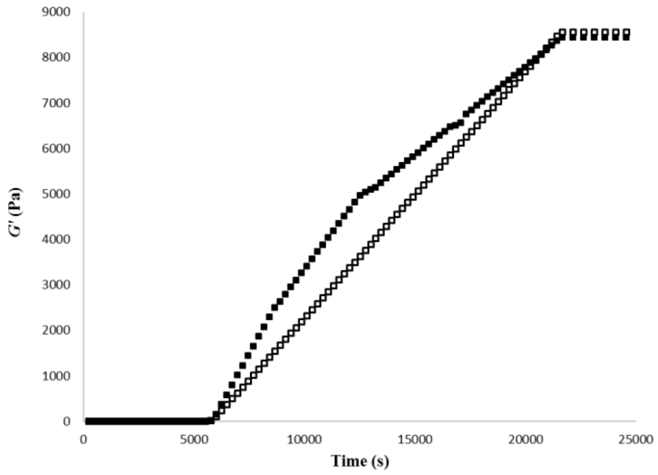

Figure S39. Time sweep rheometry of gel R_7 *ex situ* preparation method (empty □) and *in situ* preparation method (filled □).

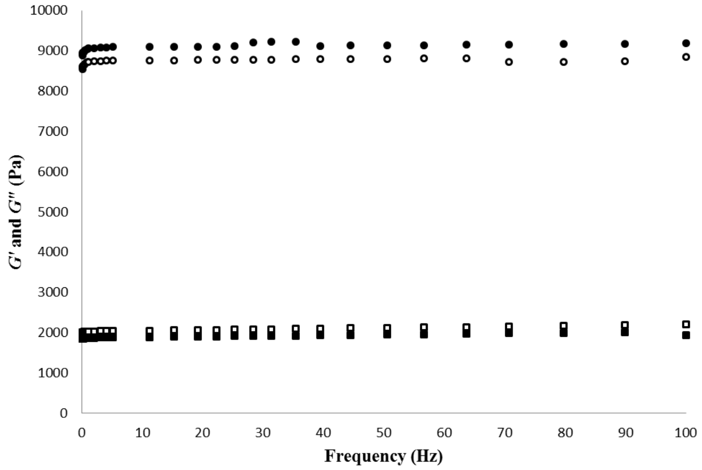

Figure S40. Frequency sweep rheometry of gel R_7 showing *ex situ* preparation method (G' = empty ○, G'' = empty □) and the *in situ* preparation method (G' = filled ○, G'' = filled □).

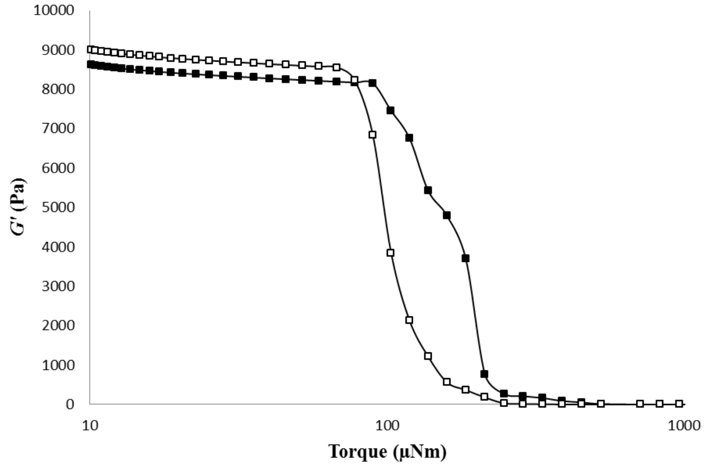

Figure S41. Stess sweep rheometry of gel R_7 showing *ex situ* preparation method (empty □) and *in situ* preparation method (filled □) (line has been added to guide the eye).

5.8 Rheological characterisation of *in situ* and *ex situ* prepared gel R_8

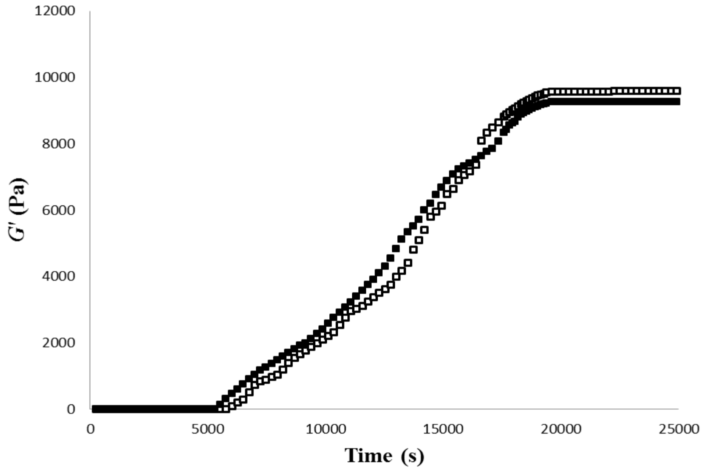

Figure S42. Time sweep rheometry of gel R_8 *ex situ* preparation method (empty □) and *in situ* preparation method (filled □).

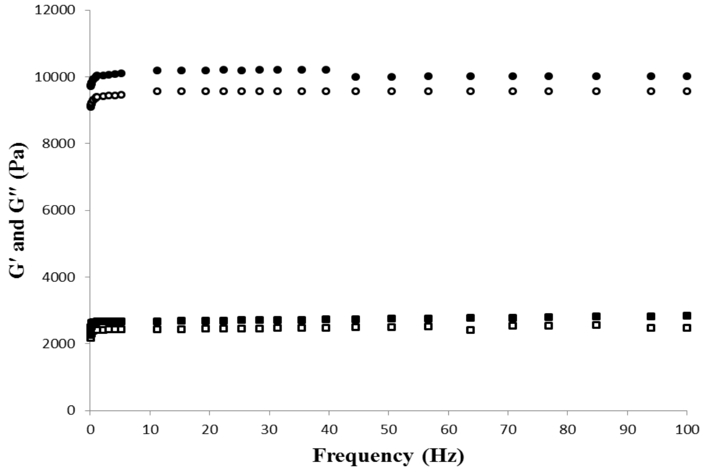

Figure S43. Frequency sweep rheometry of gel R_8 showing *ex situ* preparation method (G' = empty ○, G'' = empty □) and the *in situ* preparation method (G' = filled ○, G'' = filled □).

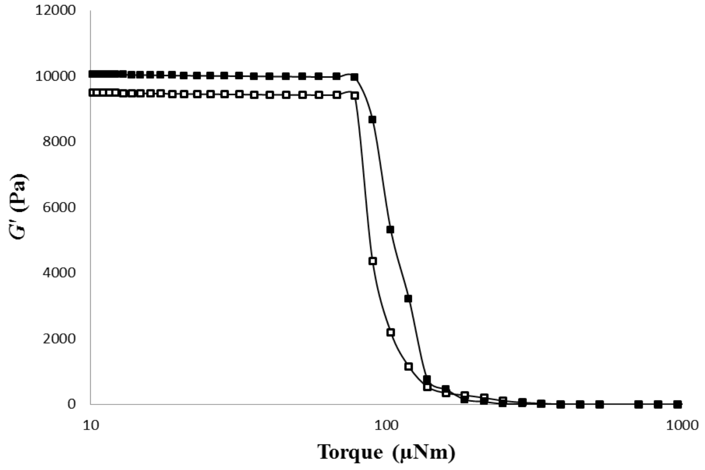

Figure S44. Stess sweep rheometry of gel R_8 showing *ex situ* preparation method (empty □) and *in situ* preparation method (filled □) (line has been added to guide the eye).

5.9 Rheological characterisation of *in situ* and *ex situ* prepared gel R_9

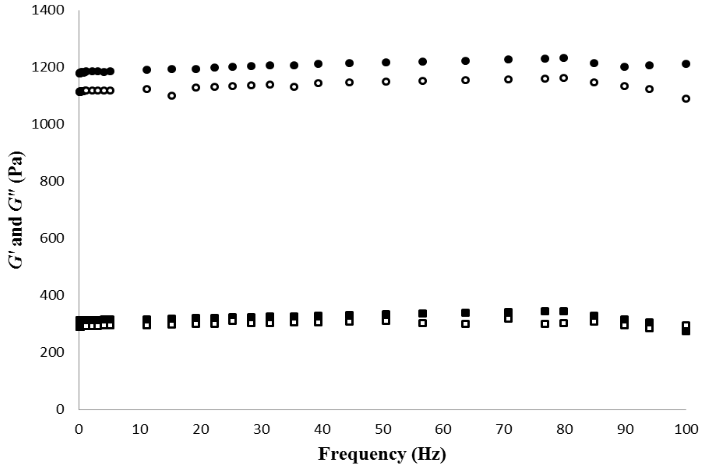

Figure S45. Frequency sweep rheometry of gel R_9 showing *ex situ* preparation method (G' = empty ○, G'' = empty □) and the *in situ* preparation method (G' = filled ○, G'' = filled □).

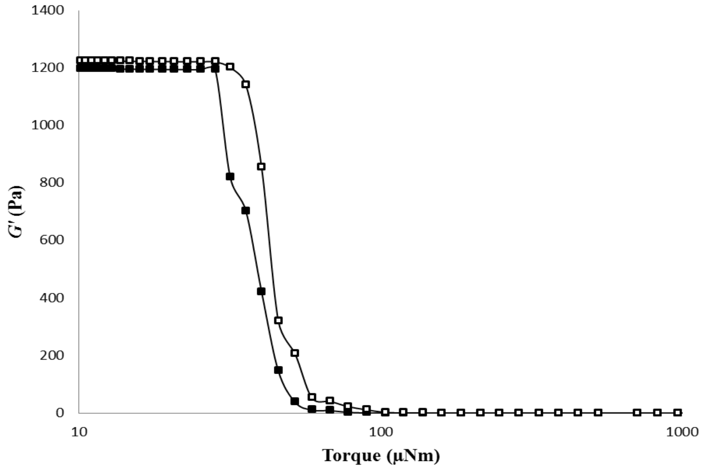

Figure S46. Stess sweep rheometry of gel R_9 showing *ex situ* preparation method (empty □) and *in situ* preparation method (filled □) (line has been added to guide the eye).

The conversion of the initial solution to each of the gels could be observed by monitoring the growth of G' over time after the addition of **GdL**. The results of these time sweep experiments make it possible to determine the Avrami constants for the formation of the gels. The Avrami constants for gels R_1 to R_9 were calculated to be 1.7, 1.6, 1.9, 1.7, 1.8, 1.7, 2.0, 1.6, respectively.

By preforming a time sweep, the data acquired allowed determination of the Avrami constant for gelation sample. The Avrami constant is a useful method of quantifying the kinetics of a phase change.[22] More recently it has been used to describe the sol to gel transition for supramolecular gelation systems.[22] The use of the Avrami equation, which is used to determine the Avrami constant, relies on the general sigmoidal profile for the extent of transformation from one phase to another.

$$\ln(1 - X_{cr}) = -kt^{D}$$

(equation 1.)

The work of Avrami is generally thought of as relating to the formation of bulk crystals, it describes how phase and ordered growth kinetics relate to physical properties such as temperature and concentration. This also means that different morphologies can arise from different growth rates.[22] Liu used the foundations laid by Avrami to develop a method where by the growth of the fibres involved in the formation of the three-dimensional supramolecular gel networks could be characterised *in situ*. The work of Liu describes the Avrami constant as the fractal dimension, however, throughout this work the term Avrami constant will be used.[22] The general form of the Avrami constant (equation 1.) where k = constant, t = time and X_{cr} = the systems crystallinity which is equal to $\phi(t)/\phi(\infty)$, where $\phi(t)$ is the volume fraction of crystal materials t, and $\phi(\infty)$ is $\phi(t)$ at $t \rightarrow \infty$. D describes the dimensions in which the bulk crystal grows, with rod-like growth described by D = 1, plate like crystals described by D = 2 and spherulite growth described by D = 3. By considering the relationship proposed by Einstein between relative viscosity and volume concentration of suspension of spheres[22] it is possible to relate X_{cr} to specific viscosity η_{sp}. Equation 2 describes specific viscosity of a system in terms of the complex viscosity η^*, and the viscosity of the medium in which the phase change is taking place, $\eta 0$.

$$\eta_{sp} = \frac{\eta^* - \eta_0}{\eta_0}$$

(equation 2.)

It is possible to define the crystallinity of the solution in terms of η^* and η_0 with respect to time as can be seen in equation 3.

$$X_{cr}(t) = \frac{\phi(t)}{\phi(\infty)} = \frac{\eta_{sp}(t)}{\eta_{sp}(\infty)} = \frac{\eta^*(t) - \eta_0}{\eta^*(\infty) - \eta_0}$$

(equation 3.)

With equation 3 it possible to observe how the viscosity of the solution as a function of time can be used to determine X_{cr}. Viscosity is a rheological property, with this in mind it is possible to use other rheological material properties to determine X_{cr}. Although gels do exhibit a relatively high degree of long-range order, they are not strictly crystalline, in most cases. With this in mind, from now on X_{cr}

will be discussed as *X,* which represents gelation as opposed to crystallinity. With a supramolecular gel's rheological characterisation the elastic modulus *G*' is representative of the materials elastic, solid-like behaviour.

$$X = \frac{G'(t)}{G'(\infty)}$$

(equation 4.)

Equation 4 shows the relationship between the elastic modulus at time *t, G*'*(t)* and it as $t \rightarrow \infty$, *G*'*(∞)*. The information required to solve for *X* can be obtained from the time sweep experiments that were performed on the gel as it set. *G*'*(∞)* is defined as the average value for the last 20 measurements of *G*'. The values of *G*'*(t)* used are up t_o ~ 10 Pa, where generally the rate of increase in *G*' is less than 0.1 Pa per measurement. The rate of increase in *G*' is important, as below 0.1 Pa per measurement, fibre growth is the key factor in the increase in *G*'*.* When this rate exceeds 0.1 Pa per measurement the branching and entangling of the existing fibres becomes an increasingly significant factor, as this marks the point at which the system moves from fibre growth to the onset of gelation.[22] With these factors in mind, the Avrami constant can be determined from equation 5.

$$ln\left(ln\left(\frac{1}{1-X}\right)\right) = ln(K) + Dln(t)$$

(equation 5.)

X can be replaced with equation 4, *K* represents a constant that is related to temperature, *t* is time and *D* is the Avrami constant itself. The *in situ* reaction system discussed here relies on the addition of GdL to set the gel meaning that the term *K* does not have to be considered. A plot of *ln*(*ln*(1/1-*X*)) against *ln*(*t*) will result in a straight line with a gradient equal to *D*.

Reference 22 refers to references in main paper.

6.0 Apparent pKas for synthesised gelators R_1 – R_9

In order to determine the apparent pKa of gelators R_1 to R_9 a series of titration experiments were performed. 1 mmol of gelator was dispersed in 15 ml of water and dissolved with the addition of NaOH. Further NaOH was added until the pH of the solution had been adjusted to approximately 10. Before aliquots of $HCl_{(aq)}$ were added and the resulting pH recorded.

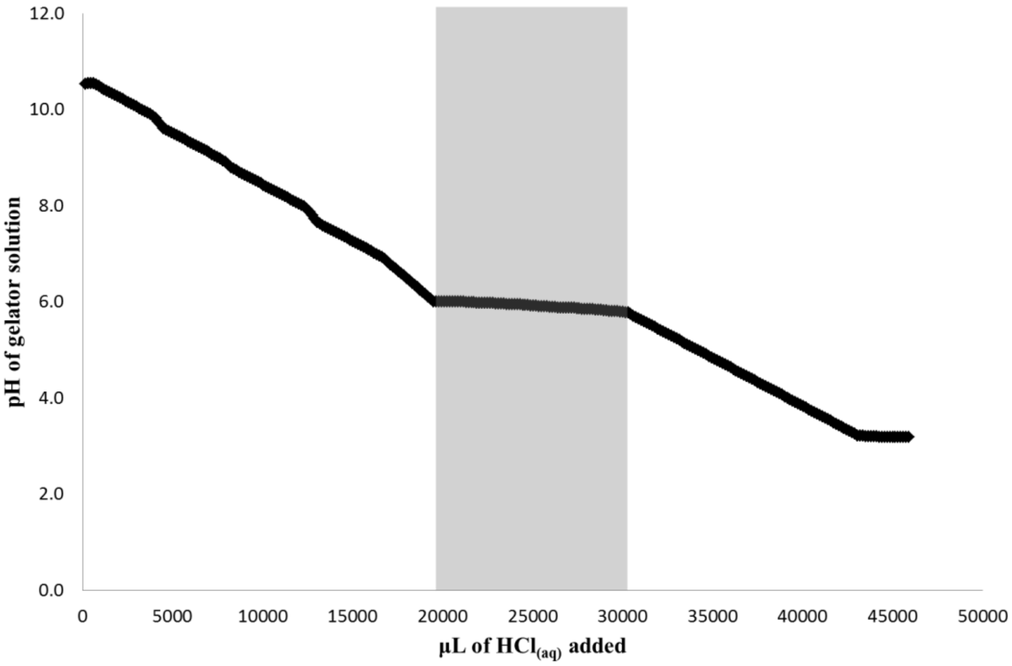

Figure S47. Apparent *pKa* determination of gelator **R1** showing and apparent *pKa* of 6.1-5.8 as taken to be the observed plateau region shown in the shaded area.

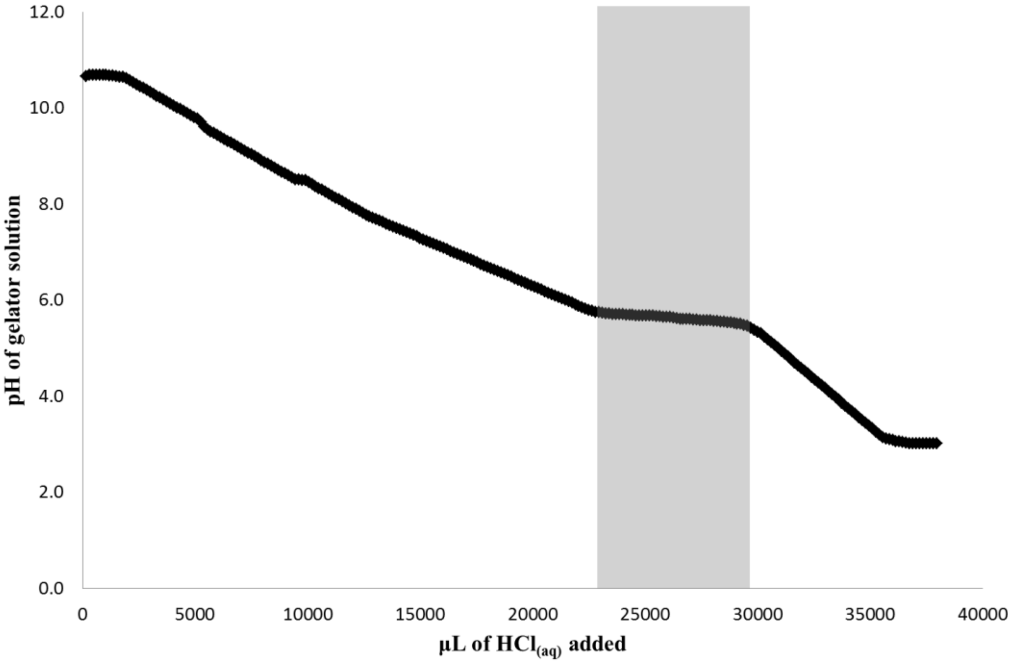

Figure S48. Apparent *pKa* determination of gelator **R2** showing and apparent *pKa* of 5.8-5.5 as taken to be the observed plateau region shown in the shaded area.

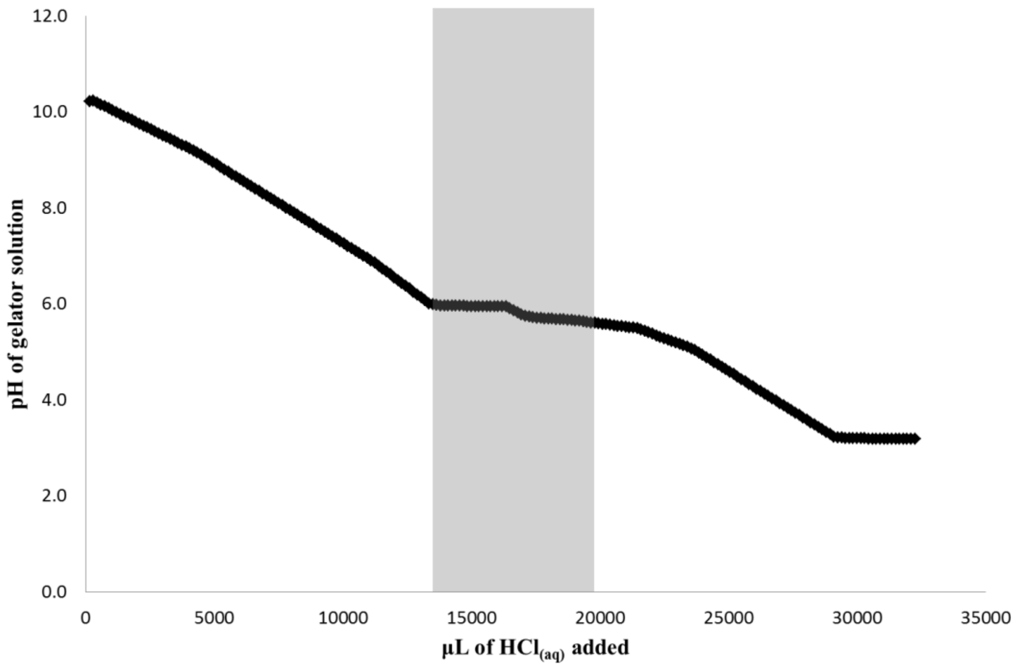

Figure S49. Apparent pK_a determination of gelator $\mathbf{R_3}$ showing and apparent pK_a of 6.1-5.7 as taken to be the observed plateau region shown in the shaded area.

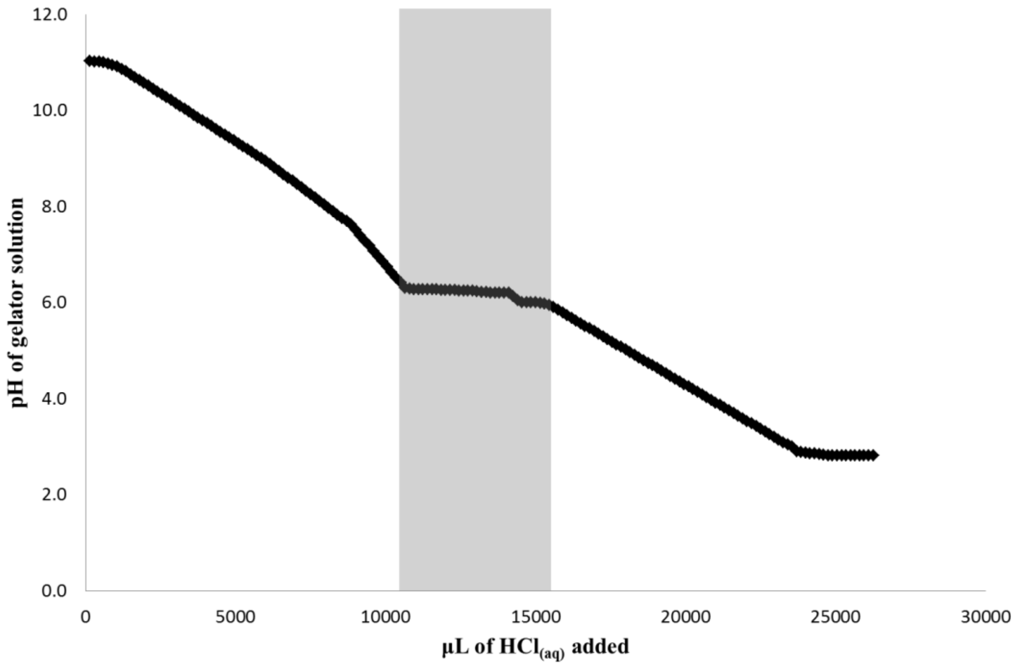

Figure S50. Apparent pK_a determination of gelator $\mathbf{R_4}$ showing and apparent pK_a of 6.5-6.2 as taken to be the observed plateau region shown in the shaded area.

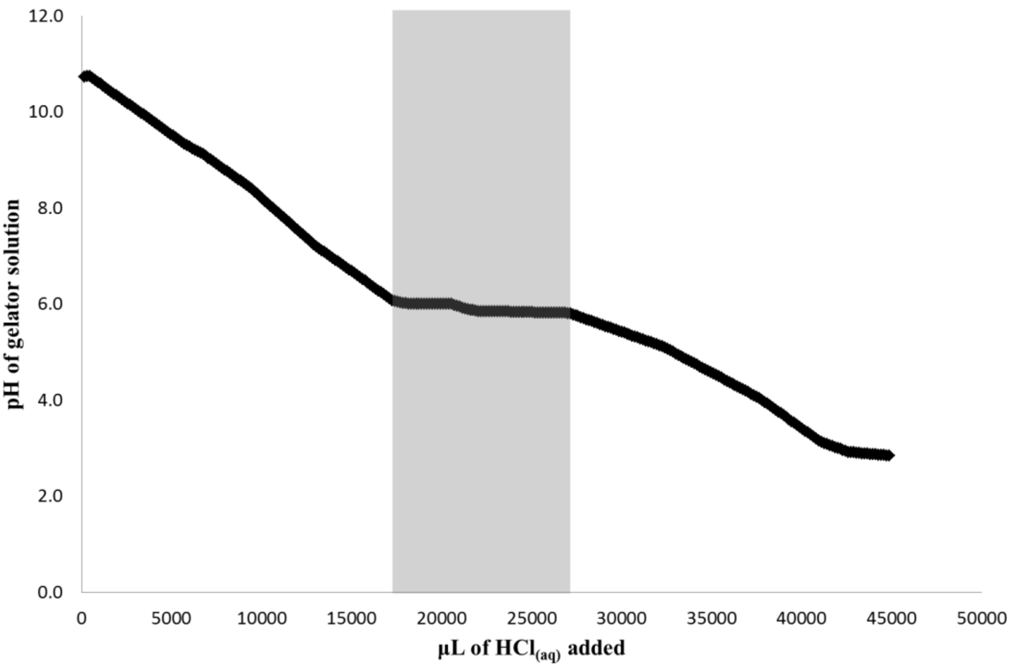

Figure S51. Apparent pK_a determination of gelator $\mathbf{R_5}$ showing and apparent pK_a of 6.1-5.8 as taken to be the observed plateau region shown in the shaded area.

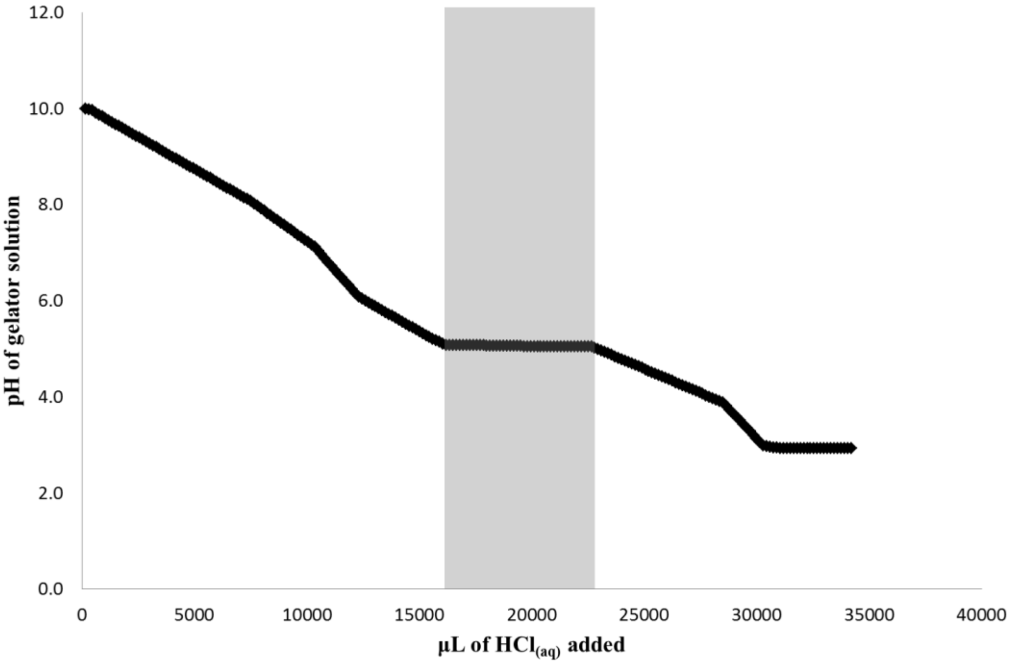

Figure S52. Apparent pK_a determination of gelator $\mathbf{R_6}$ showing and apparent pK_a of 5.2-5.0 as taken to be the observed plateau region shown in the shaded area.

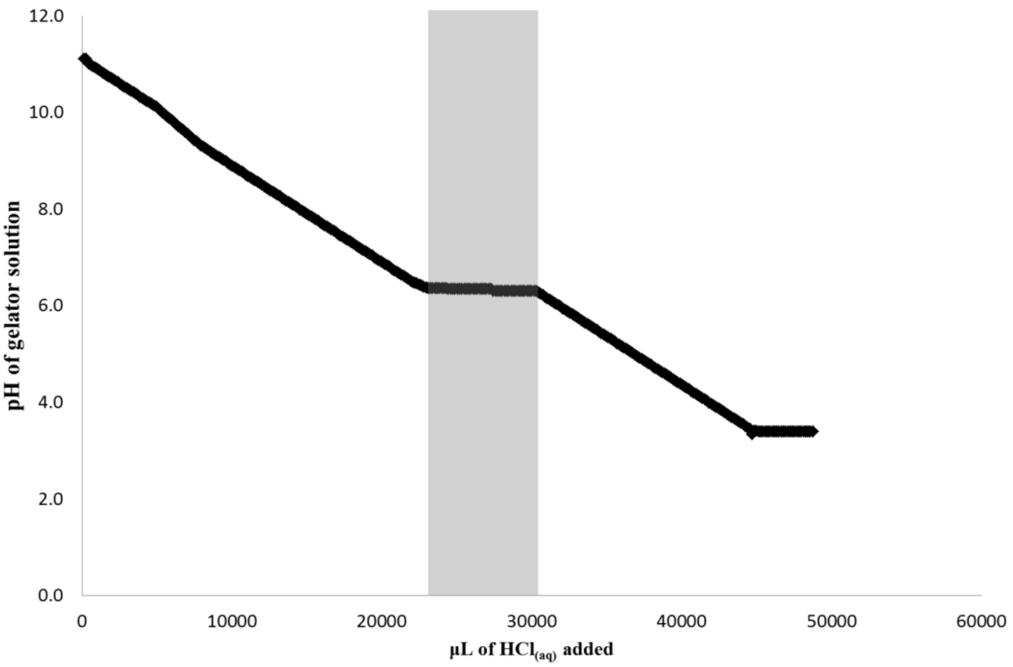

Figure S53. Apparent pK_a determination of gelator $\mathbf{R_7}$ showing and apparent pK_a of 6.5-6.3 as taken to be the observed plateau region shown in the shaded area.

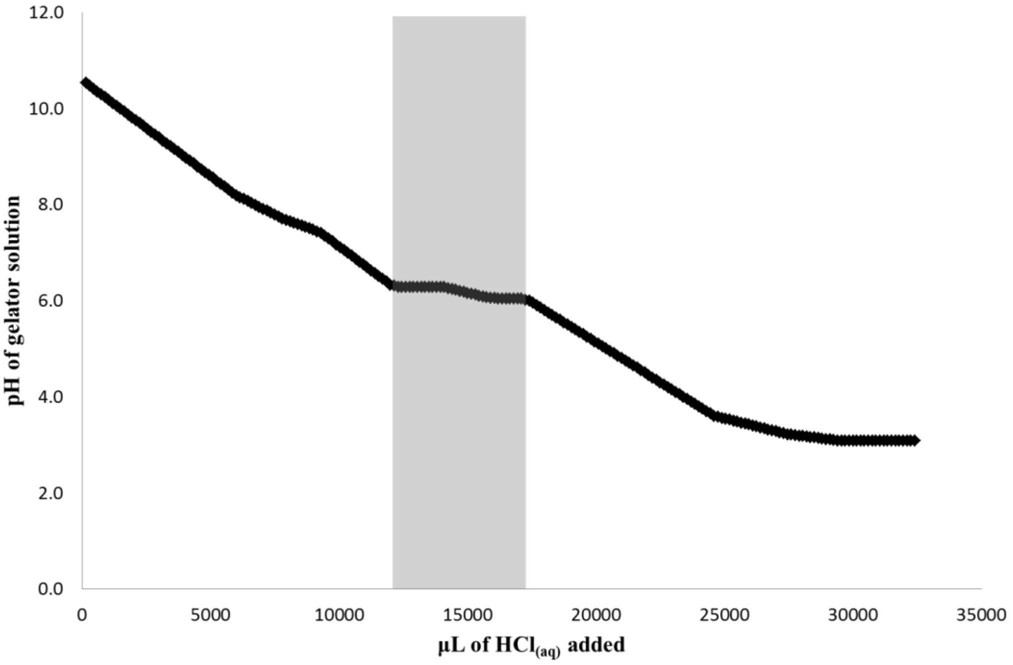

Figure S54. Apparent pK_a determination of gelator $\mathbf{R_8}$ showing and apparent pK_a of 6.4-6.0 as taken to be the observed plateau region shown in the shaded area.

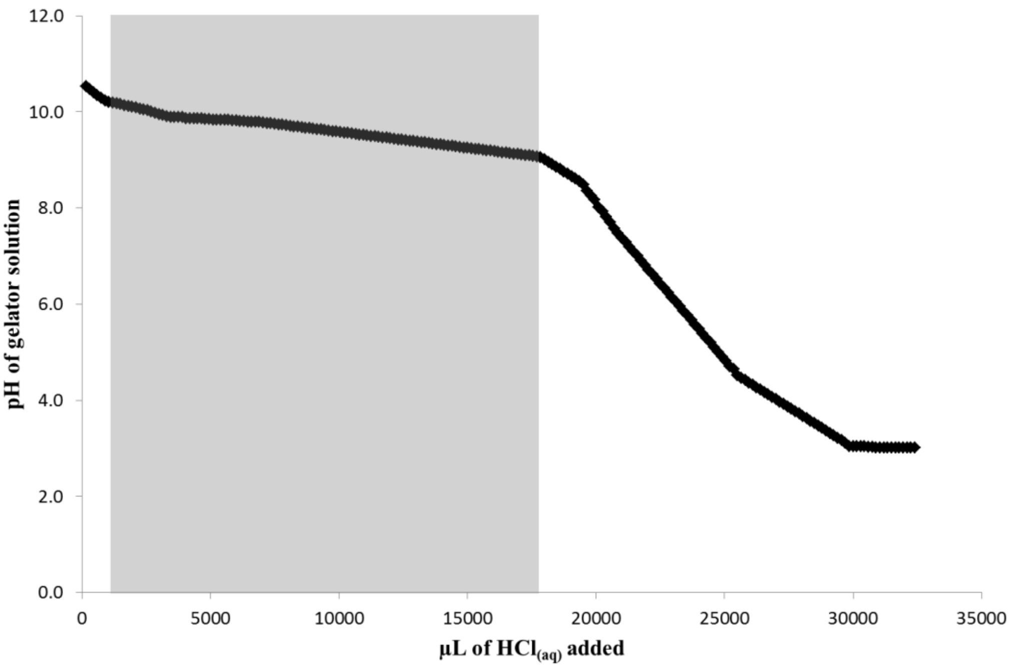

Figure S55. Apparent *pKa* determination of gelator **R9** showing and apparent *pKa* of 9.9-9.0 as taken to be the observed plateau region shown in the shaded area.

7.0 PXRD Patterns for dried gel samples $R_1 – R_9$

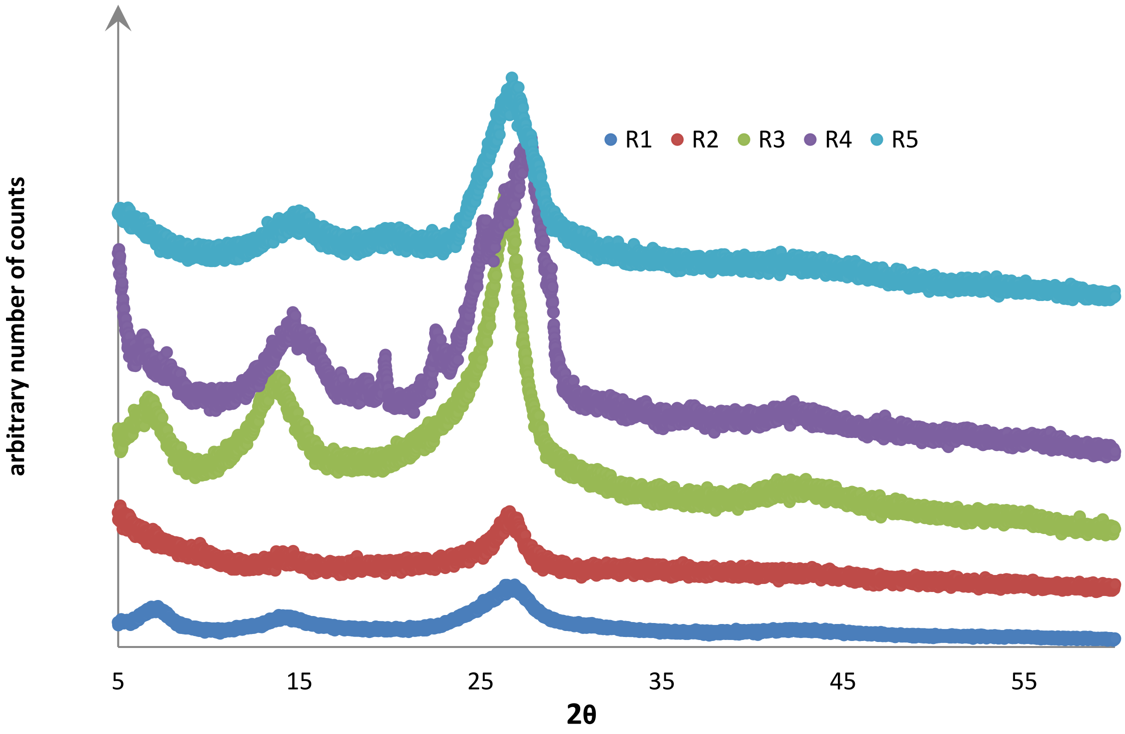

Figure S56. PXRD patterns for samples produced from dried gels R_1, R_2, R_3, R_4 and R_5 recorded between 5 – 60 ° for 30 minutes.

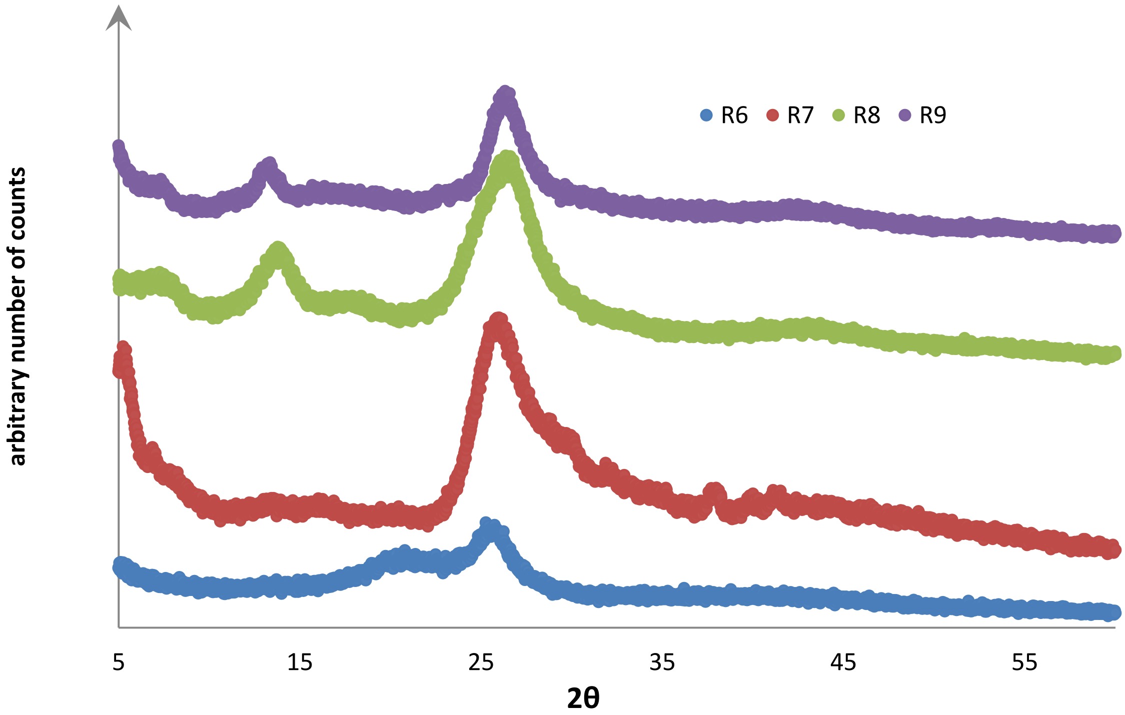

Figure S57. PXRD patterns for samples produced from dried gels R_6, R_7, R_8 and R_9 recorded between 5 – 60 ° for 30 minutes.

7.1 PXRD pattern of non gelator R_{10}

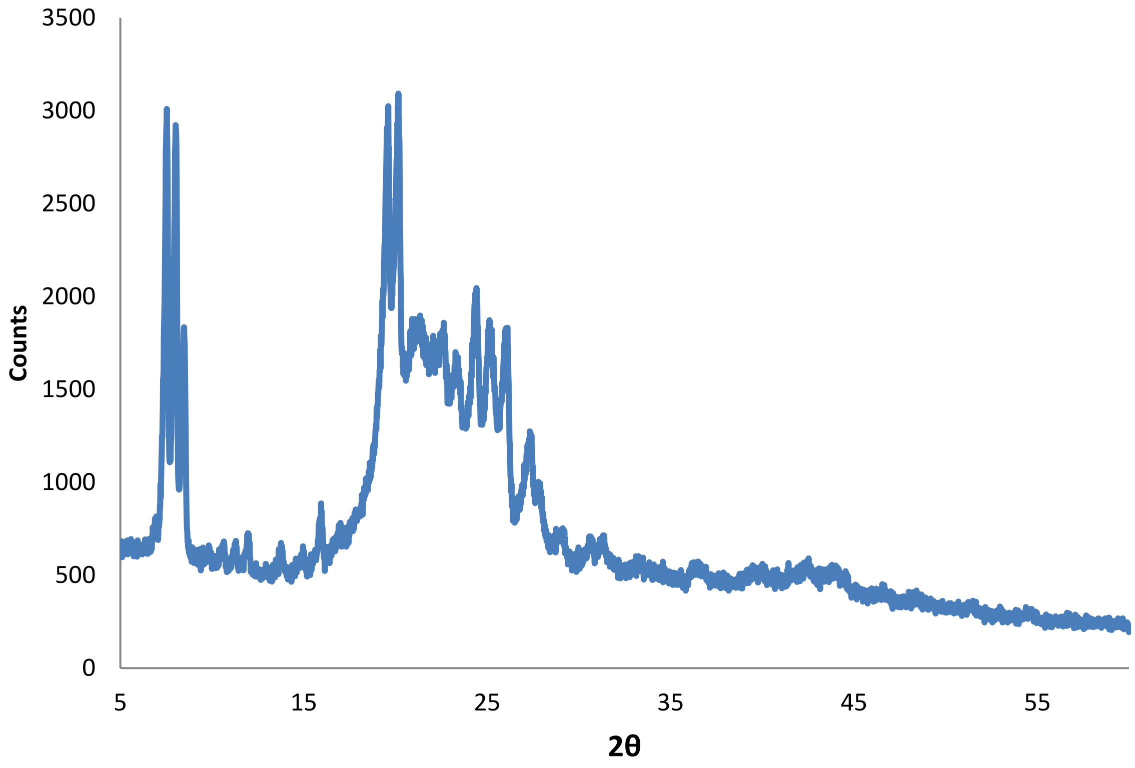

Figure S58. PXRD patterns for samples produced from precipitate collected from an attempted gelation of R_{10} using **GdL**. Recorded between 5 – 60 º for 30 minutes.

8.0 Analysis of mixed gelators systems.

8.1 UV-vis transmission data recorded for various gels of R_1 and R_6 mixtures

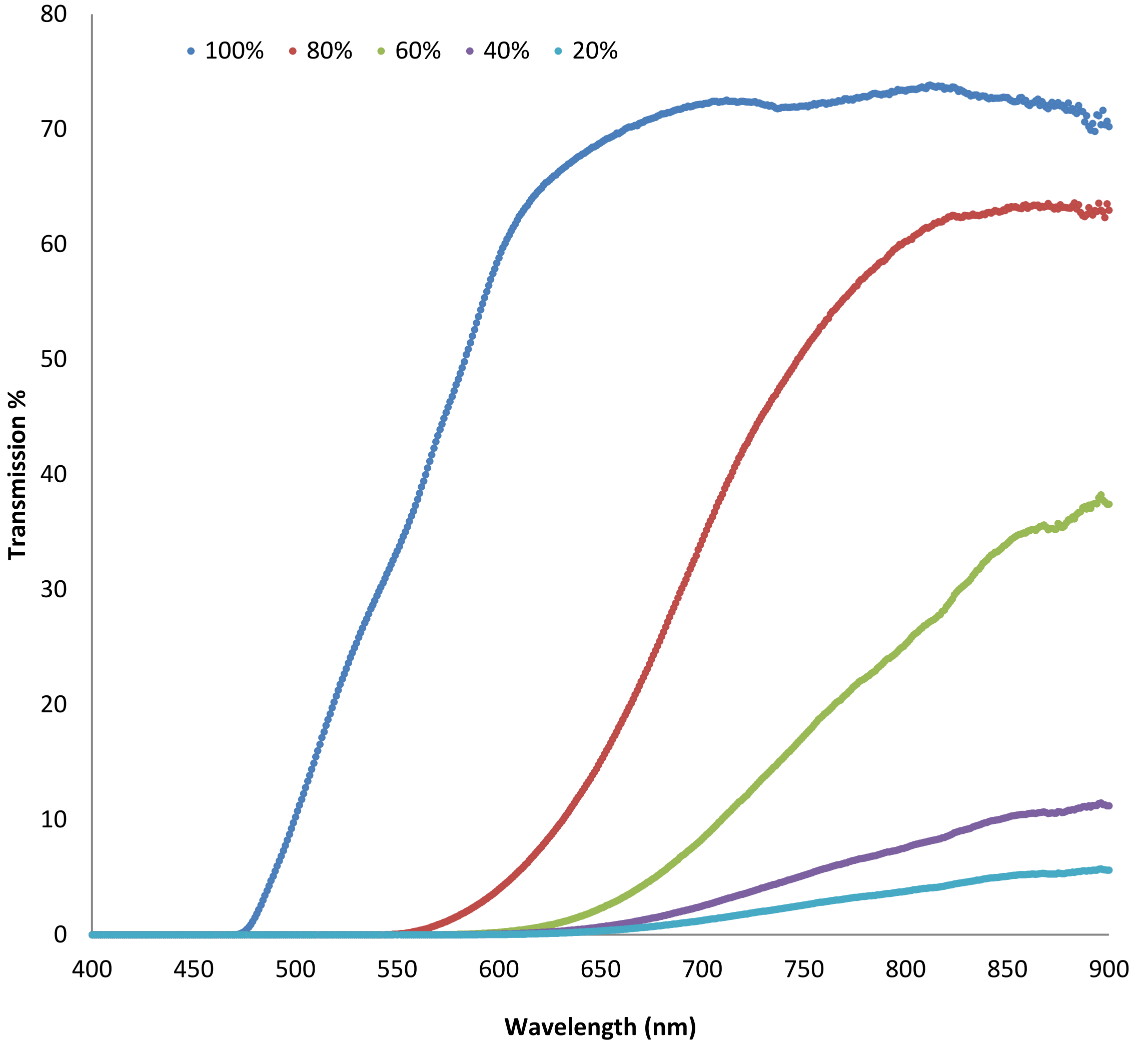

Figure S59. UV-vis transmission data showing a decrease in transmission with a reduction in R_6 relative to the total concentration of gelator (R_1 + R_6) from 100 - 20 % R_6 in 20 % steps.

8.2 PXRD analysis gels of R_1 and R_6 mixtures

Figure S60. PXRD patterns for samples produced from dried mix gel systems(100 % R_1, 75 % R_1 : 25 % R_6, 50 % R_1 : 50 %

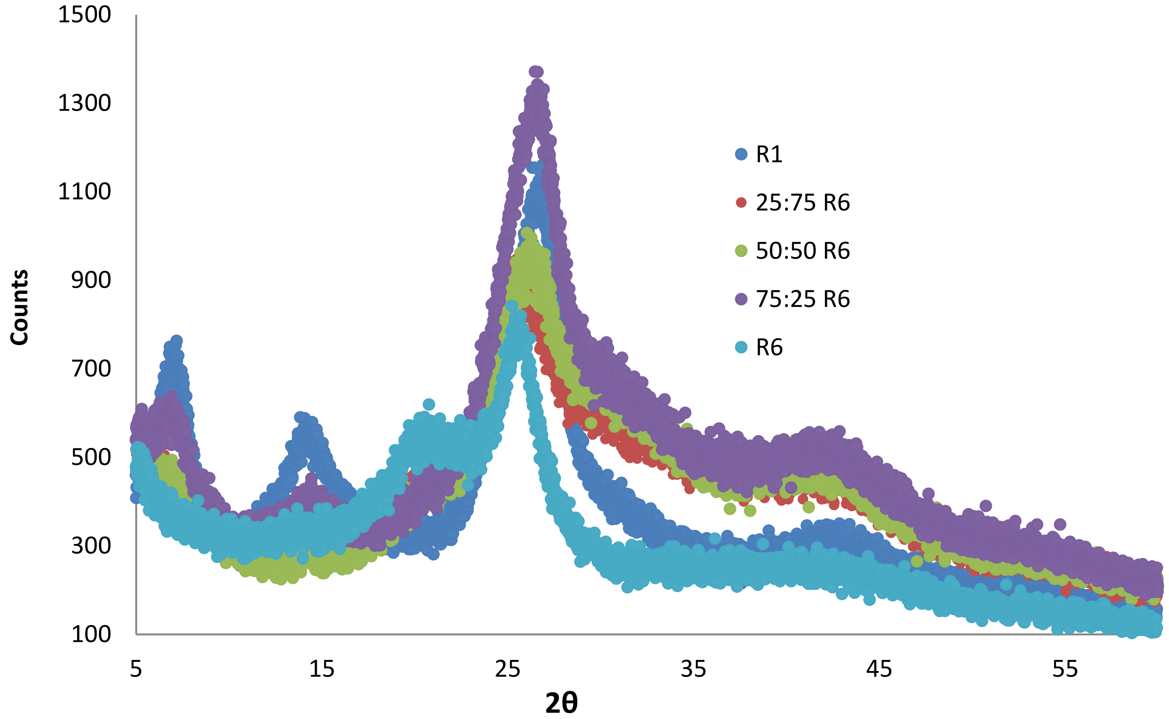

R_6, 25 % R_1 : 75 % R_6, 100 % R_6) recorded between 5 – 60 ° for 30 minutes.

Figure S61. Expansion of **Figure S60**. between 22 ° and 29 °.

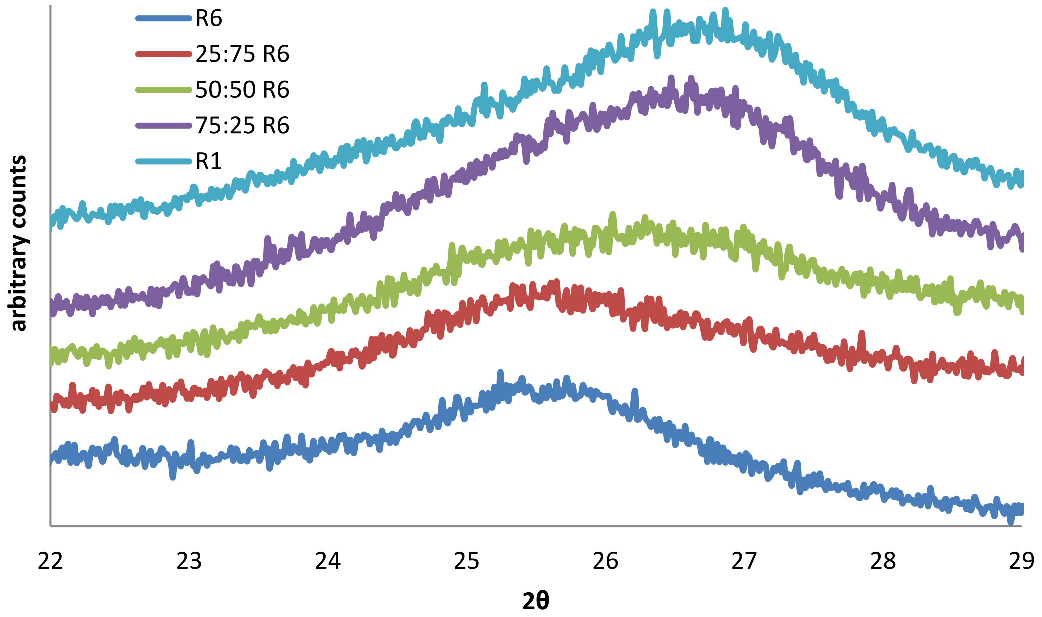

Table S2. Analysis of PXRD data for mixed (R_1 and R_6) gel systems.

Gel	Angle (°)	*d* spacing
100 % R_1	25.2435	3.53
75 % R_1 : 25 % R_6	25.6055	3.47
50 % R_1 : 50 % R_6	26.0544	3.42
25 % R_1 : 75 % R_6	26.4743	3.36
100 % R_6	26.8652	3.32

As can be seen in table S2 the *d* spacing decreases with increasing concentration of R_6 relative to the total concentration of the gelator.

8.3 Morphology determined by SEM for gels of R_1 and R_6 mixtures.

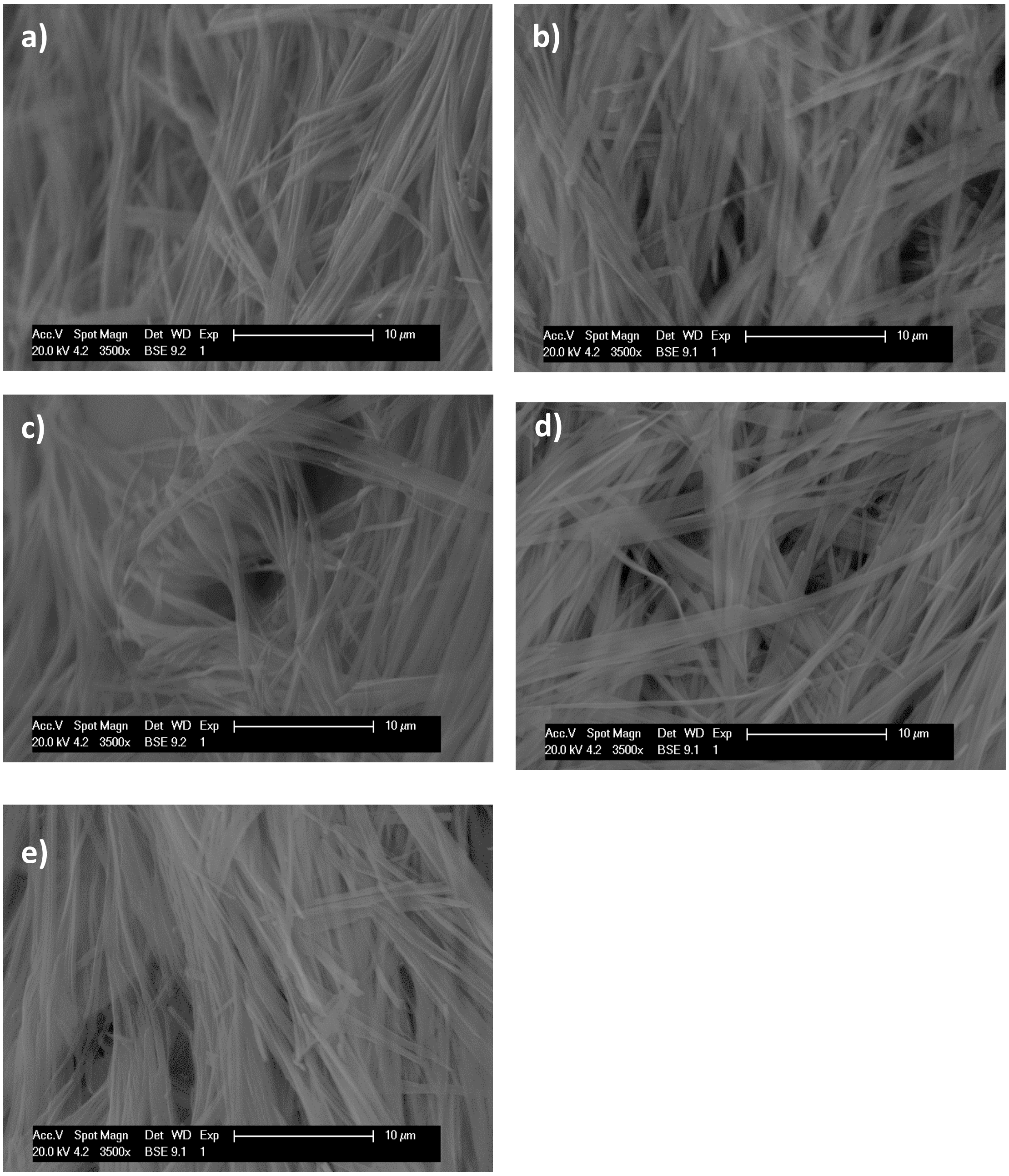

Figure S62. SEM analysis showing the consistency of morphology for gels set using various molar ratios of R_1 and R_6. a) R_1 100 : 0 R_6. b) R_1 75 : 25 R_6. c) R_1 50 : 50 R_6. d) R_1 25 : 75 R_6. e) R_1 0 : 100 R_6.

8.4 Apparent pKa of R_1 and R_6 mixtures.

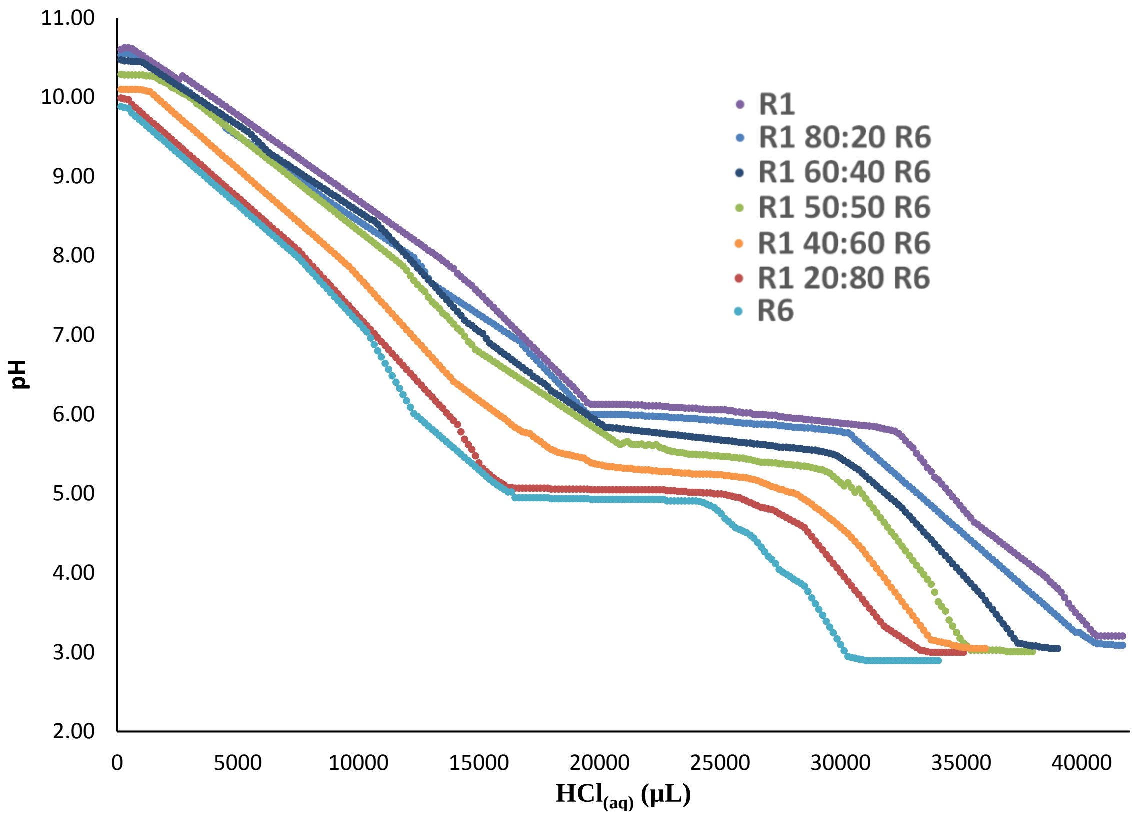

Figure S63. Apparent pK_a determination for mixtures of R_1 and R_6 from 100% R_1 to 100% R_6 in 20% increments relative to the total concentration of gelator molecule 80 mg in 20 ml of water. Apparent pK_a was determined with constant rapid stirring to prevent gelation.

The apparent pK_a is defined as the plateau region where the change in the pH of the solution is minimal. As can be seen for the R_1 : R_6 mixtures there is a gradual and constant reduction in the apparent pK_a from the value of 100% R_1 to that of 100% R_6. This shows that both gelators influence the apparent pK_a region collectively allowing co-assembly to occur given the correct gelation conditions.

8.5 NMR of R_1 : R_6 gelator co-assembly process

A solution was created by dissolving 25 µmol of R_1 and 25 µmol of R_6 in D_2O with the addition of NaOH and a spectra was obtained. 10 µmol of **GdL** was then added and a spectra was recorded every five minutes for a total of two hours and over a range of 6 – 9 ppm. Examination of the integrations of peaks (determined against the internal standard of 4-aminobenzoic acid) that relate to the aromatic protons of both **R1** and **R6** allowed the plot shown in Figure S64 to be produced. The initial measurement at time zero (before the addition of GdL) was used as the reference with which to normalise the data.

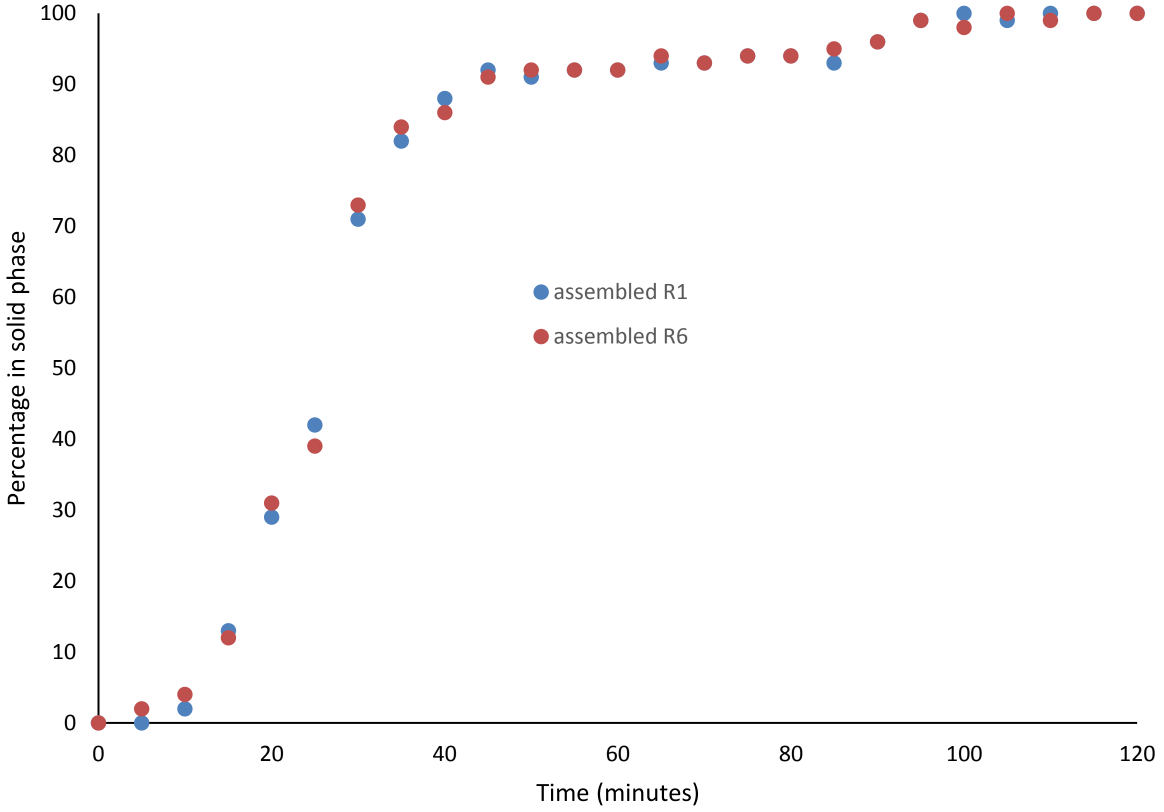

Figure S64. Co-assembly over time of gelators R_1 and R_6 as determined by ^1H NMR.

8.6 PXRD analysis of xerogels of R_1 and R_{10} 50:50 mixture

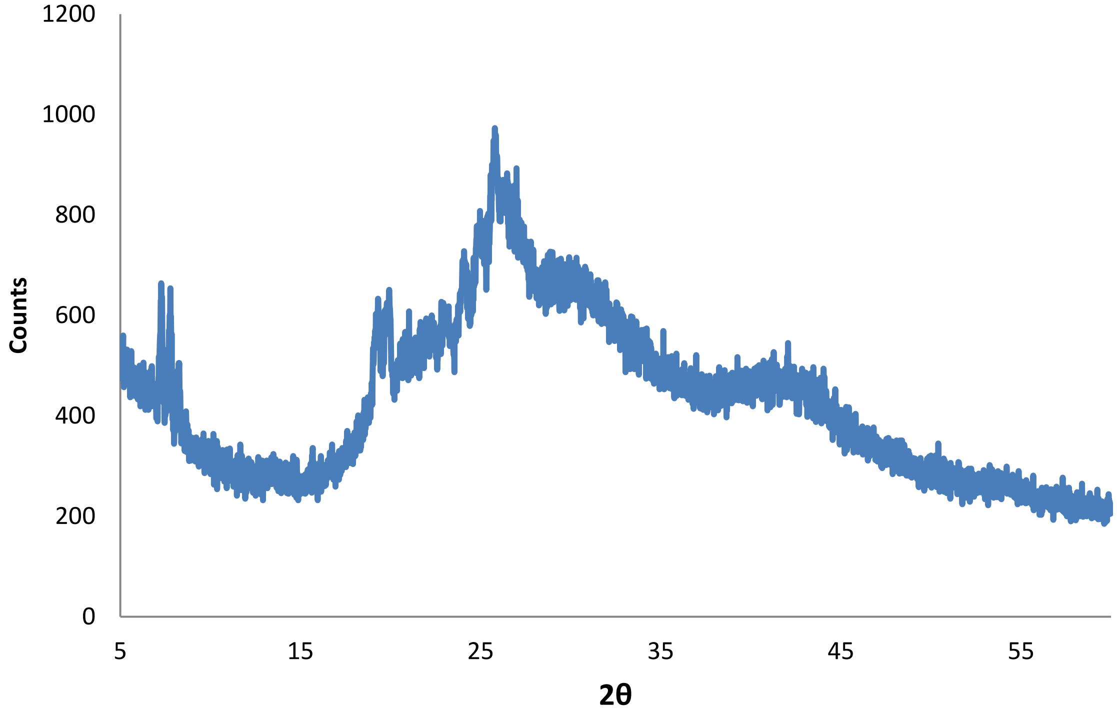

Figure S65. PXRD pattern of $R_1 : R_{10}$ (50 : 50) xerogel.

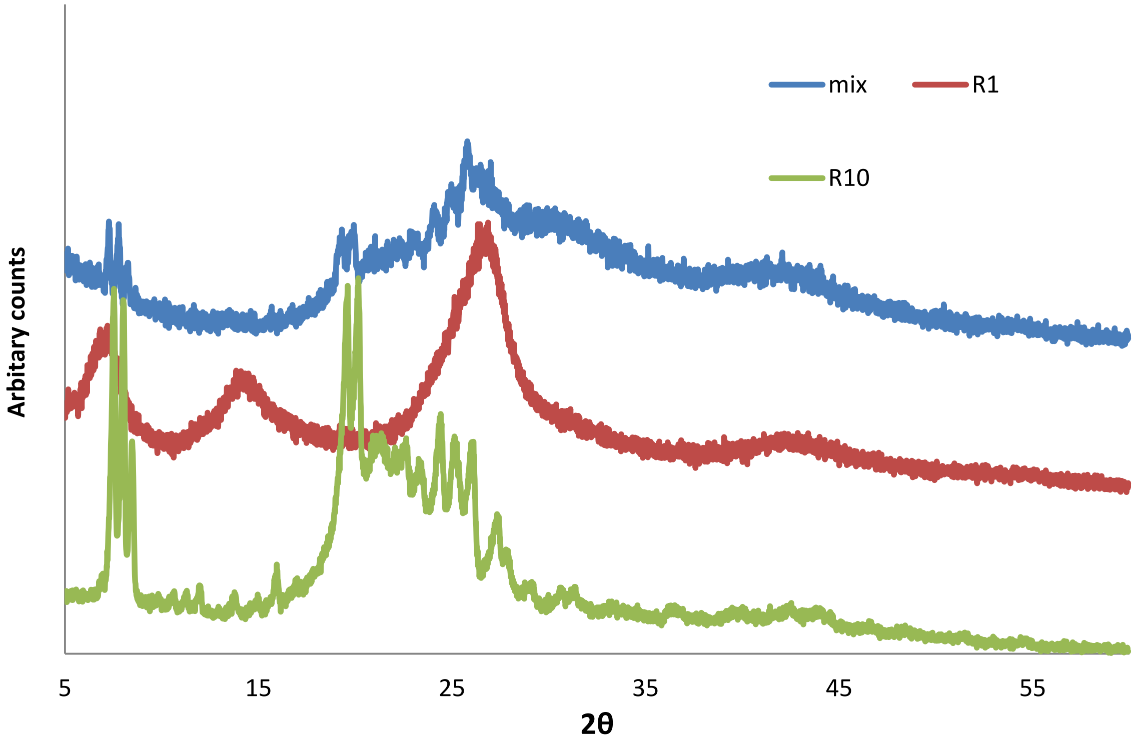

Figure S66. Pure R_{10} compound as synthesised (green). Pure R_1 xerogel (red). $R_1 : R_{10}$ (50 : 50) xerogel (blue) highlights the self-sorting ability of the 50:50 $R_1 : R_{10}$ gel into gel phase R_1 and precipitate R_{10}.

8.7 Apparent pK_a of R_1 and R_{10} mixture

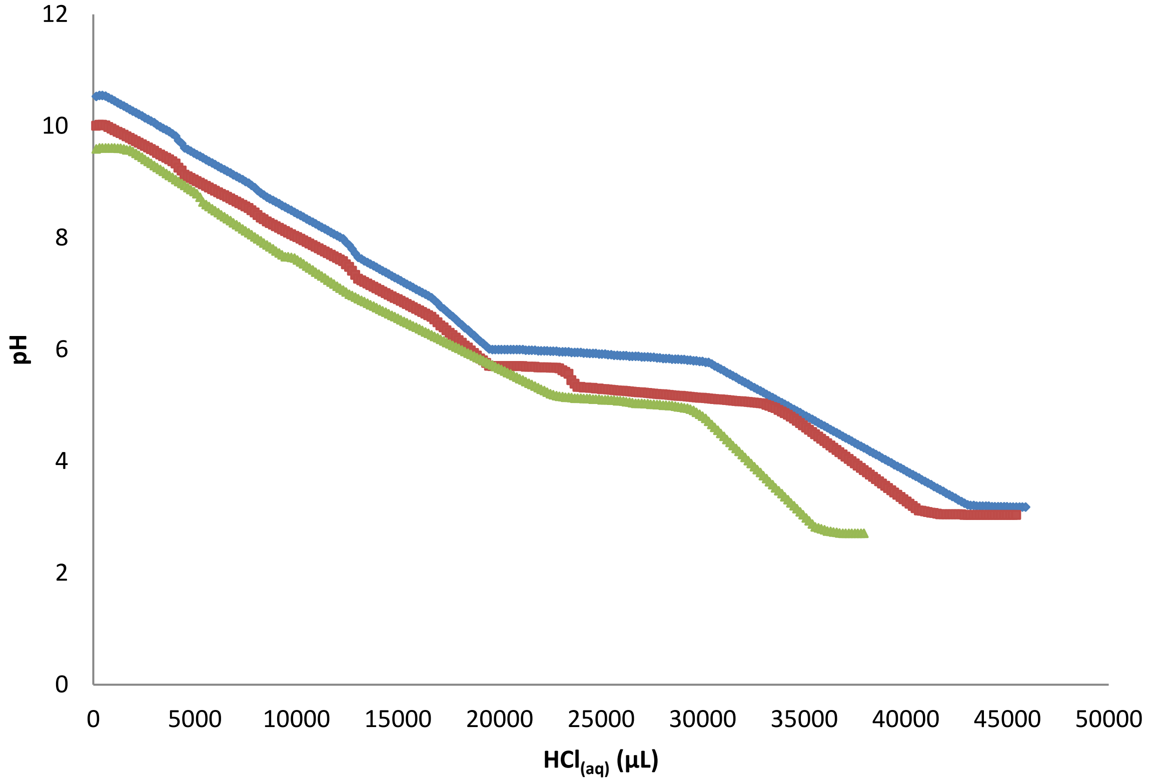

Figure S67. Apparent pK_a determination for mixturs of R_1 and R_{10} (50 : 50) mix (red). Pure R_1 (blue). Pure R_{10} (green). Step in the plateau of R_1 and R_{10} (50 : 50) mix shows a stepwise self-sorting co-assembly process.

8.8 NMR of R_1 : R_{10} gelator self-sorting process

A solution was created by dissolving 25 µmol of R_1 and 25 µmol of R_{10} in D_2O with the addition of NaOH and a spectra was obtained. 10 µmol of **GdL** was then added and a spectra was recorded every five minutes. The same procedure described for the R_1 and R_6 mixed samples, section 7.5, was utilised.

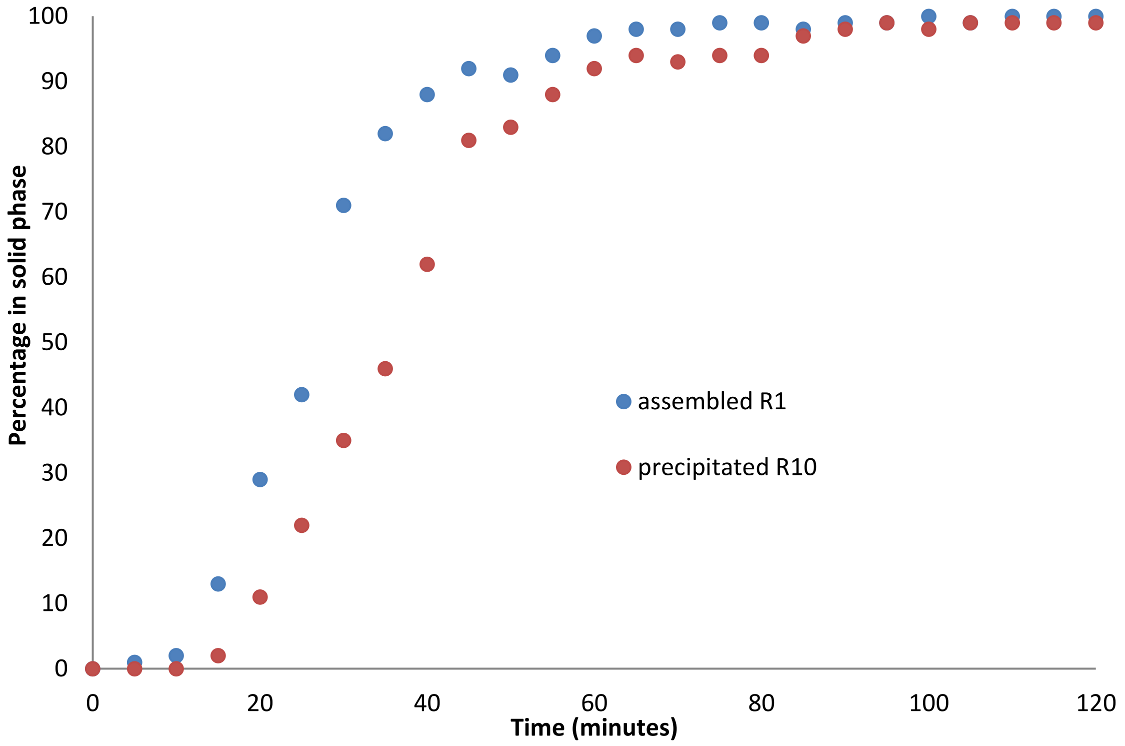

Figure S68. Self-sorting experiment of gelators R_1 and R_{10} over time as determined by ^1H NMR. The two compounds are not disappearing through self-assembly at the same rate as the pH is changing, nor have they had the same initiation point (5 mins vs 15mins).

8.9 HPLC of R_1 : R_6 gelator process

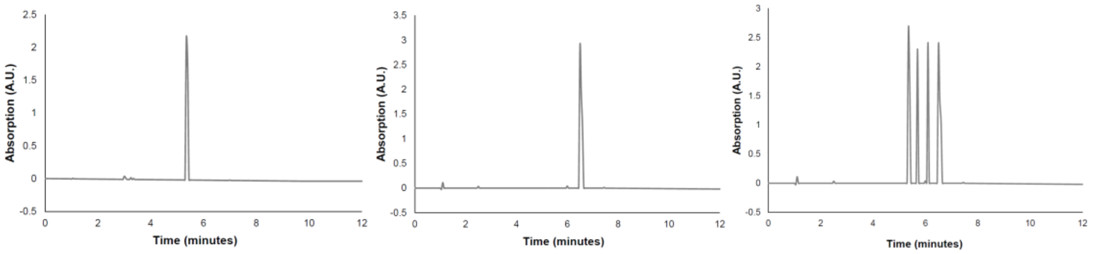

Figure S69. From left to right, HPLC trace for *ex situ* synthesised compound **R1**, with a retention time of 323 seconds recorded; *ex situ* synthesised compound **R6**, with a retention time of 391 seconds recorded; and *in situ* reaction mixture containing **R1** amine, **R6** amine and ***II***. All components were dissolved in water at pH 8, with the HPLC analysis performed after 16 hours.

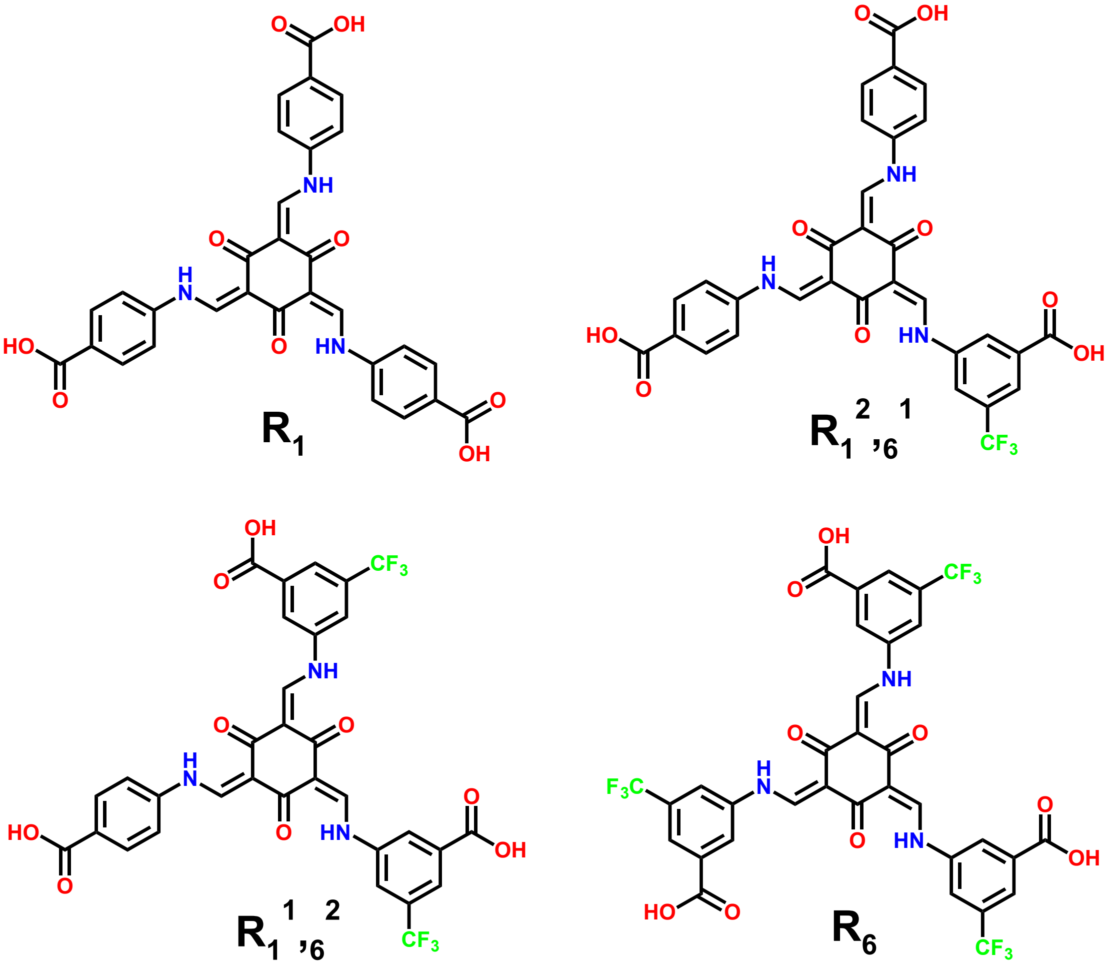

Figure S70. All possible reaction products for the *in situ* $\mathbf{R_1}$ + $\mathbf{R_6}$ + ***II***, including the gelators $\mathbf{R_1}$, $\mathbf{R_6}$, and products composed of mixed amines $\mathbf{R_1{}^2{}_{,6}{}^1}$ and $\mathbf{R_1{}^1{}_{,6}{}^2}$.

9.0 Computational Calculations

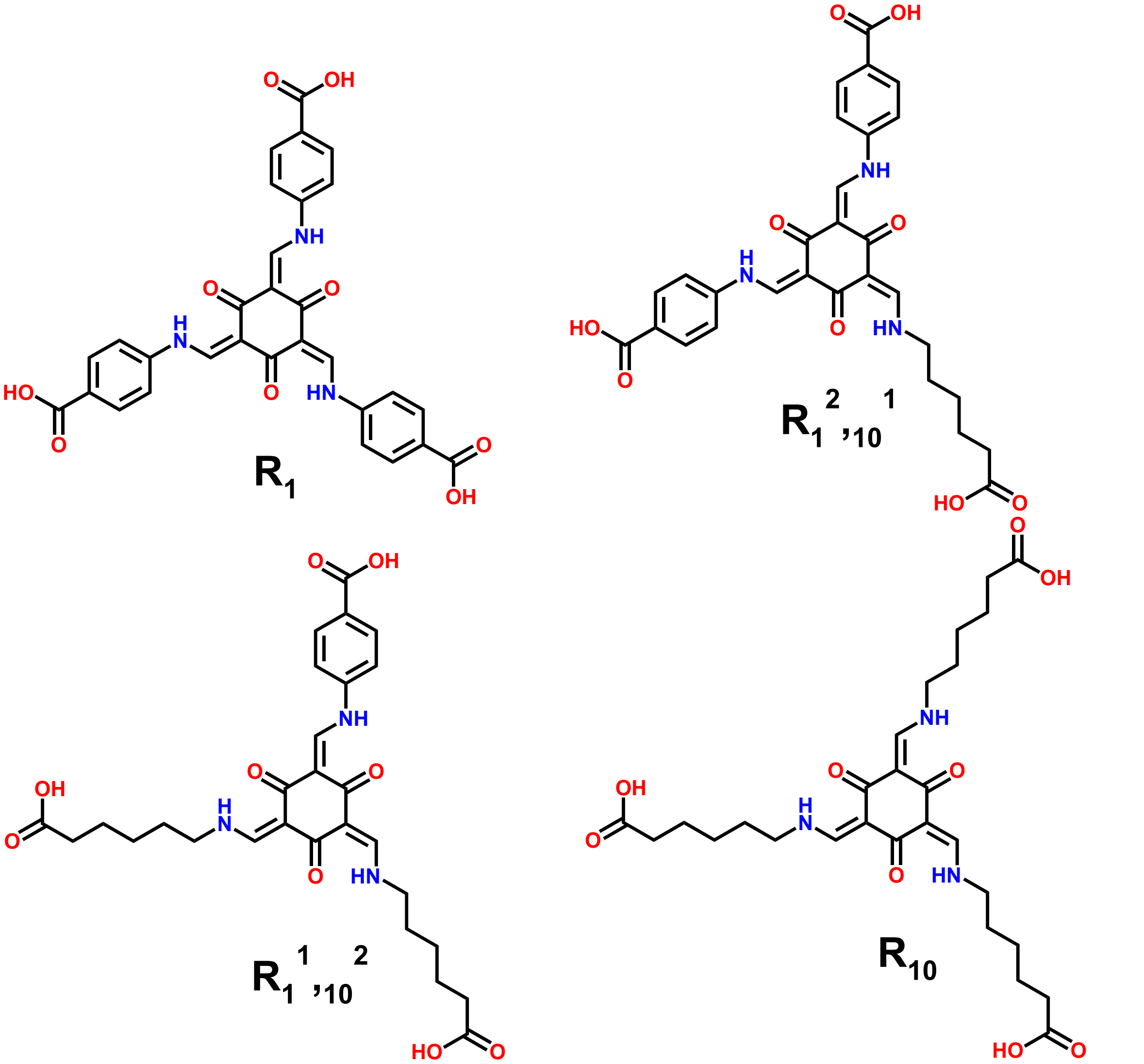

Figure S71. All possible reaction products for the *in situ* **R**$_1$ + **R**$_{10}$ + ***II***, including the known molecules **R**$_1$ and **R**$_{10}$, and products composed of mixed amines **R**$_1{}^2$,$_{10}{}^1$ and **R**$_1{}^1$,$_{10}{}^2$. Chemical analyses highlight a chemical self-sorting of these compounds resulting in only the pure **R**$_{10}$ and **R**$_1$ species during *in situ* reactions. **R**$_{11}$ variants would be the methyl derivatives of the four compounds shown above.

Table S3. Computed electronic and zero point corrected energy differences between the C_3 and C_s conformations of various tripodal ketoenamine molecules. All energies are in kcal mol^{-1}.

	B3LYP		B97-D	
Structure	Electronic	Zero Point Correct.	Electronic	Zero Point Correct.
3**R**$_1$	1.041	1.170	1.032	1.199
2**R**$_1$: 1**R**$_{10}$	1.145	1.296	1.146	1.227
1**R**$_1$: 2**R**$_{10}$	0.875	0.981	0.939	0.950

3**R**$_{10}$	0.911	1.158	0.916	1.178
2**R**$_1$: 1**R**$_{11}$	1.156	1.313	1.076	1.302
1**R**$_1$: 2**R**$_{11}$	0.942	1.041	0.943	1.115
3**R**$_{11}$	1.034	1.091	1.029	1.046
2**R**$_1$: 1**R**$_6$	1.061	1.242	1.031	1.304
1**R**$_1$: 2**R**$_6$	1.232	1.461	1.241	1.547
3**R**$_6$	1.321	1.568	1.326	1.684

Table S4. Computed electronic and zero point corrected reaction energetics, using the B3LYP functional, for the formation of various tripodal keto-enamine systems. All values reported pertain to the C_3 conformation and in terms of kcal mol^{-1}. **R**$_{11}$ being the methyl derivative to allow for reduction of the conformational flexibility of the calculated compounds.

	Electronic			Zero Point Corrected		
Structure	1st	2nd	3rd	1st	2nd	3rd
3**R**$_1$	6.928	7.149	7.316	4.809	4.950	5.133
2**R**$_1$: 1**R**$_{11}$	6.928	7.149	1.316	4.809	4.950	-0.565
1**R**$_1$: 2**R**$_{11}$	0.527	1.561	7.664	-1.390	-0.291	5.542
3**R**$_{11}$	0.527	1.561	2.474	-1.390	-0.291	0.617
2**R**$_1$: 1**R**$_6$	6.928	7.149	6.913	4.809	4.950	4.752
1**R**$_1$: 2**R**$_6$	6.690	6.807	7.190	4.563	4.576	5.019
3**R**$_6$	6.690	6.807	6.865	4.563	4.576	4.625

Table S5. Computed electronic and zero point corrected reaction energetics, using the B97-D functional, for the formation of various tripodal keto-enamine systems. All values reported pertain to the C_3 conformation and are in kcal mol^{-1}.

	Electronic			Zero Point Corrected		
Structure	1st	2nd	3rd	1st	2nd	3rd
3**R**$_1$	4.801	4.986	5.128	2.625	2.784	2.714
2**R**$_1$: 1**R**$_{11}$	4.801	4.986	-0.031	2.625	2.784	-1.940
1**R**$_1$: 2**R**$_{11}$	-0.834	0.284	5.555	-2.780	-1.526	3.452
3**R**$_{11}$	-0.834	0.284	1.258	-2.780	-1.526	-0.540
2**R**$_1$: 1**R**$_6$	4.801	4.986	4.764	2.625	2.784	2.375
1**R**$_1$: 2**R**$_6$	4.542	4.602	5.049	2.373	2.342	2.771
3**R**$_6$	4.542	4.602	4.697	2.373	2.342	2.434

Table S6. Computed electronic and zero point corrected energy for the keto-enol tautomerisation for the three-subunit systems in the C_3 conformation. All values are in kcal mol^{-1}.

	B3LYP		B97-D	
Structure	Electronic	Zero Point Correct.	Electronic	Zero Point Correct.
3$\mathbf{R}_1$	-14.289	-12.181	-14.937	-11.546
2$\mathbf{R}_1$: 1$\mathbf{R}_{11}$	-13.986	-11.972	-14.519	-11.417
1$\mathbf{R}_1$: 2$\mathbf{R}_{11}$	-13.507	-11.505	-13.973	-11.037
3$\mathbf{R}_{11}$	-12.742	-10.725	-13.101	-10.111
2$\mathbf{R}_1$: 1$\mathbf{R}_6$	-13.374	-11.472	-14.159	-10.987
1$\mathbf{R}_1$: 2$\mathbf{R}_6$	-12.609	-10.762	-13.405	-10.439
3$\mathbf{R}_6$	-11.852	-9.965	-12.758	-9.855
